# Preclinical Efficacy of a Lipooligosaccharide Peptide Mimic Candidate Gonococcal Vaccine

**DOI:** 10.1128/mBio.02552-19

**Published:** 2019-11-05

**Authors:** Sunita Gulati, Michael W. Pennington, Andrzej Czerwinski, Darrick Carter, Bo Zheng, Nancy A. Nowak, Rosane B. DeOliveira, Jutamas Shaughnessy, George W. Reed, Sanjay Ram, Peter A. Rice

**Affiliations:** a Department of Medicine, Division of Infectious Diseases and Immunology, University of Massachusetts Medical School, Worcester, Massachusetts, USA; b AmbioPharm, Inc., North Augusta, South Carolina, USA; c Peptides International Inc., Louisville, Kentucky, USA; d Infectious Diseases Research Institute, Seattle, Washington, USA; Emory University School of Medicine

**Keywords:** *Neisseria gonorrhoeae*, vaccine, peptide, antibody function, experimental infection, immunization/vaccine

## Abstract

The global spread of multidrug-resistant strains of Neisseria gonorrhoeae constitutes a public health emergency. With limited antibiotic treatment options, there is an urgent need for development of a safe and effective vaccine against gonorrhea. Previously, we constructed a prototype vaccine candidate comprising a peptide mimic (mimitope) of a glycan epitope on gonococcal lipooligosaccharide (LOS), recognized by monoclonal antibody 2C7. The 2C7 epitope is (i) broadly expressed as a gonococcal antigenic target in human infection, (ii) a critical requirement for gonococcal colonization in the experimental setting, and (iii) a virulence determinant that is maintained and expressed by gonococci. Here, we have synthesized to >95% purity through a relatively facile and economical process a tetrapeptide derivative of the mimitope that was cyclized through a nonreducible thioether bond, thereby rendering the compound homogeneous and stable. This vaccine candidate, called TMCP2, when administered at 0, 3, and 6 weeks to BALB/c mice at either 50, 100 or 200 μg/dose in combination with glucopyranosyl lipid A-stable oil-in-water nanoemulsion (GLA-SE; a Toll-like receptor 4 and T_H_1-promoting adjuvant), elicited bactericidal IgG and reduced colonization levels of gonococci in experimentally infected mice while accelerating clearance by each of two different gonococcal strains. Similarly, a 3-dose biweekly schedule (50 μg TMCP2/dose) was also effective in mice. We have developed a gonococcal vaccine candidate that can be scaled up and produced economically to a high degree of purity. The candidate elicits bactericidal antibodies and is efficacious in a preclinical experimental infection model.

## Abstract (precis)

Neisseria gonorrhoeae has become resistant to most antibiotics. The incidence of gonorrhea is also sharply increasing. A safe and effective antigonococcal vaccine is urgently needed. Lipooligosaccharide (LOS), the most abundant outer membrane molecule, is indispensable for gonococcal pathogenesis. A glycan epitope on LOS that is recognized by monoclonal antibody (MAb) 2C7 (called the 2C7 epitope) is expressed almost universally by gonococci *in vivo*. Previously, we identified a peptide mimic (mimitope) of the 2C7 epitope, which when configured as an octamer and used as an immunogen, attenuated colonization of mice by gonococci. Here, a homogenous, stable tetrameric derivative of the mimitope, when combined with a T_H_1-promoting adjuvant and used as an immunogen, also effectively attenuates gonococcal colonization of mice. This candidate peptide vaccine can be produced economically, an important consideration for gonorrhea, which affects socioeconomically underprivileged populations disproportionately, and represents an important advance in the development of a gonorrhea vaccine.

## INTRODUCTION

Neisseria gonorrhoeae, the causative agent of the sexually transmitted disease gonorrhea, has become resistant to almost every antibiotic in clinical use. The combination of ceftriaxone and azithromycin is the current first-line treatment for gonorrhea recommended by the Centers for Disease Control and Prevention (CDC) (1). However, resistance to each of these antibiotics have been reported in several countries (2–5), which further limits treatment options. In addition, the number of cases of gonorrhea is increasing rapidly: in 2018, 583,405 cases of gonorrhea were reported to the CDC, a 63% increase since 2014 and an 82.6% increase since the historic low in 2009 (https://www.cdc.gov/std/stats18/gonorrhea.htm). The World Health Organization estimated that 86.9 million cases of gonorrhea occurred globally in 2016 (63). Therefore, there is an urgent need for safe and effective vaccines against gonorrhea to prevent the global spread of multidrug-resistant organisms.

Lipooligosaccharide (LOS) is the most abundant molecule expressed on the gonococcal surface and contributes significantly to its pathogenesis. Natural and experimental gonococcal infection in humans induces the development of anti-LOS antibodies (6–9), which may provide some protection against reinfection (6). An epitope on LOS that is expressed by almost every gonococcal isolate *in vivo* is defined by binding of a monoclonal antibody (MAb) called 2C7 (7). Binding of MAb 2C7 requires the presence of lactose extensions simultaneously from heptose I (HepI) and HepII (10); MAb 2C7 can bind to LOS even in the presence of glycan extensions beyond lactose from HepI (11).

Elaboration of the 2C7 epitope by N. gonorrhoeae requires expression of a phase-variable LOS glycosyltransferase gene called *lgtG* (12), which adds the proximal Glc residue at the 3-position of HepII (expression of the 2C7 epitope is completed by *lgtE*, which is constitutively expressed and adds Gal to HepII-linked Glc). Absence of HepII-linked lactose (and therefore the complete 2C7 structure/epitope) significantly attenuates gonococcal infection in the mouse cervicovaginal colonization model (13–15). 2C7 expression therefore may be an important virulence factor that enhances or is required for gonococcal survival *in vivo* in humans. Furthermore, we recently showed that lactose from HepII can be capped with sialic acid (15), which decreases complement deposition (15) and engages members of the Siglec family of proteins (16) to limit the host inflammatory response to infection. These functions of sialic acid on lactose expressed from HepII, together with a fitness requirement for the 2C7 structure, may explain widespread expression of the 2C7 LOS epitope on N. gonorrhoeae (7, 15), which is displayed by most gonococci, including 95% of minimally passaged N. gonorrhoeae clinical isolates in Boston, MA (7), and in 100% of clinical isolates in Nanjing, China (15).

Previously, we showed that a peptide mimic of the 2C7 epitope (a mimitope), when configured as an octameric “multiantigen peptide” (Octa-MAP) (17) and administered to BALB/c mice with Sigma MPL adjuvant, elicited bactericidal antibodies (IgG Ab responses were T_H_1 skewed) and also attenuated gonococcal vaginal colonization in the experimental model of infection (13). In this report, we evaluate the candidacy of the 2C7 mimitope that has now been configured as a stable, highly pure tetrapeptide and administered with glucopyranosyl lipid A (GLA) formulated in a stable oil-in-water nanoemulsion (SE), an adjuvant approved for use in humans. This study is an important step in the development of a safe, effective, and economical gonococcal vaccine.

## RESULTS

### Characterization of a lead vaccine candidate, TMCP2.

**FIG 1:**
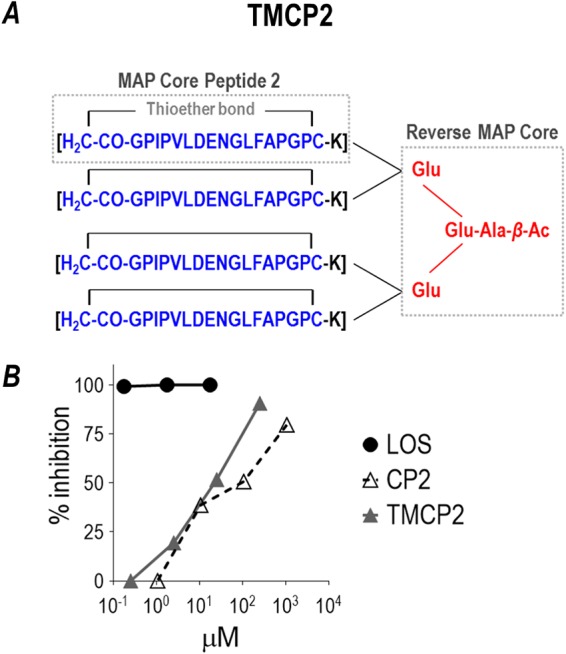
Structure and characterization of TMCP2. (A) Chemical structure of TMCP2, the 2C7 mimitope peptide configured as a tetrameric multiantigenic peptide (MAP). The 2C7 mimitope is indicated in blue. Cyclization is maintained through a thioether bond. The MAP core is indicated in red and links to each cyclic peptide through a lysine (K) residue. A schematic of the steps in the synthesis of TMCP2 is shown in Fig. S1. (B) Inhibition of MAb 2C7 binding to solid-phase-affixed (coated) gonococcal LOS by monomeric cyclic peptide 2 (CP2), TMCP2, and nominal LOS (control). MAb 2C7 (0.04 μg/ml) was added to microtiter wells coated with LOS purified from gonococcal strain 15253 in the presence of increasing concentrations of CP2, TMCP2, or LOS (positive control for 100% inhibition). The *y* axis shows the percentage of inhibition of MAb 2C7 binding (residual binding) to immobilized LOS in the presence of the peptide (TMCP2 or CP2) or soluble LOS relative to binding of MAb 2C7 alone to immobilized LOS.

Initial synthesis of our vaccine candidate, a multivalent antigenic peptide that encompassed 8 individual copies of the mimitope of the 2C7 epitope (termed PEP1) onto a lysine core (13), yielded a heterogenous compound because reversible disulfide bonding occurred between the terminally located Cys residues on PEP1—internally within the same molecule and externally to other like molecules, resulting in a pattern of extreme molecular weight (MW) heterogeneity (see Table S1a in the supplemental material). In order to synthesize a stable homogenous compound potentially suitable for clinical use, several strategies were undertaken, further documented in the supplemental material (Text S1, Table S1, Table S2, Fig. S1A to C). A final stable compound that circularized the mimitope via a stable nonreducible (covalent) thioether bond between the terminal cysteines in linear PEP1 formed the cyclized peptide, renamed monomeric cyclic peptide 2 (CP2). CP2 was linked to each of four lysines in a scaffold where –NH_2_ groups on lysines, in turn, were linked to –COOH groups on glutamate molecules in a core that formed the basis of the tetrameric CP2 structure, called TMCP2 (Fig. 1A). The purity of TMCP2 determined by reverse-phase high-performance liquid chromatography (RP-HPLC) separation showed a major peak (99% of the total area) at 13.155 min (Fig. S1D). The identity of TMCP2 was verified with positive electrospray ionization (ESI) time-of-flight mass spectrometry (TOF MS ES+) (Fig. S1E). We used enzyme-linked immunosorbent assay (ELISA) to measure inhibition of binding (residual binding) of MAb 2C7 that had first been reacted with increasing concentrations of the peptides, to immobilized gonococcal LOS (Fig. 1B). We observed 50% inhibition of MAb 2C7 binding to immobilized 15253 LOS in the presence of 25 μM and 107 μM TMCP2 and CP2, respectively; the approximately 4-fold lower molar concentration of TMCP2 (compared to CP2) required to achieve 50% inhibition is consistent with its tetrameric nature. The overall higher avidity and maintenance of the 4:1 molar ratio in binding experiments suggested that linking CP2 to the MAP core did not alter antigenicity of the final compound. Purified LOS, used as a positive control, completely inhibited MAb 2C7 binding to immobilized LOS.

10.1128/mBio.02552-19.1 TEXT S1 Supplemental text. Download Text S1, PDF file, 0.1 MB. Copyright © 2019 Gulati et al. 2019 Gulati et al. This content is distributed under the terms of the Creative Commons Attribution 4.0 International license.

10.1128/mBio.02552-19.2 FIG S1 (A) Inhibition of MAb 2C7 binding to solid-phase-affixed (coated) gonococcal LOS by: TMCP2, TMCP3, and TMCP4 (see Text S1 and Table S1k for description of peptides) and nominal LOS (control). (B) Anti-LOS antibody elicited by immunization of BALB/c mice with TMCP2, TMCP3, and TMCP4 (see Text S1 and Table S1k for description of peptides). (C) Serum bactericidal activity (SBA) of mouse antisera raised against tetra-MAP vaccine candidates (50 μg/dose at weeks 0, 3, 6, 9, and 12) with Sigma MPL adjuvant given via the subcutaneous (SC) or intramuscular (IM) routes (see Fig. S2 for immunization details). (D) The purity of TMCP2 determined by high-performance reverse-phase separation. (E) Positive electrospray ionization time-of-flight mass spectrometry (TOF MS ES+) of purified TMCP2. Download FIG S1, PDF file, 0.4 MB. Copyright © 2019 Gulati et al. 2019 Gulati et al. This content is distributed under the terms of the Creative Commons Attribution 4.0 International license.

10.1128/mBio.02552-19.6 TABLE S1 Summary of peptides synthesized and tested. Download Table S1, PDF file, 0.4 MB. Copyright © 2019 Gulati et al. 2019 Gulati et al. This content is distributed under the terms of the Creative Commons Attribution 4.0 International license.

10.1128/mBio.02552-19.7 TABLE S2 Chemical structures of peptides tested. Download Table S2, PDF file, 0.1 MB. Copyright © 2019 Gulati et al. 2019 Gulati et al. This content is distributed under the terms of the Creative Commons Attribution 4.0 International license.

### Choice of GLA-SE as the lead adjuvant.

Because Sigma MPL cannot be used clinically, we tested TMCP2 with three additional adjuvants with properties similar to Sigma MPL: (i) glucopyranosyl lipid adjuvant (GLA) in a stable oil-in-water nanoemulsion (SE) (GLA-SE), (ii) GLA-liposome QS21 (GLA-LSQ), and (iii) GLA-aqueous formulation (GLA-AF) (see Fig. S2 in the supplemental material). Sigma MPL adjuvant was the control to bridge data from prior experiments. Alum (Alhydrogel) and the stable oil-in-water nanoemulsion (SE) were also tested. TMCP2 plus GLA-SE or GLA-SQ yielded the highest anti-LOS IgG titers, similar to those elicited by Sigma MPL; GLA-AF gave intermediate titers, while alum and SE yielded low titers (Fig. S2A).

10.1128/mBio.02552-19.3 FIG S2 Choice of GLA-SE as the adjuvant for tetra-MAP vaccine candidate TMCP2. Download FIG S2, PDF file, 0.2 MB. Copyright © 2019 Gulati et al. 2019 Gulati et al. This content is distributed under the terms of the Creative Commons Attribution 4.0 International license.

In light of the importance of complement-dependent killing for the activity of MAb 2C7 *in vivo* (18), serum bactericidal assays were performed to assess functionality of immune antibodies. During the course of the antigen optimization and adjuvant selection studies (performed between mid-2016 and early 2018), we noted that mouse sera when heat inactivated (56°C for 30 min to deplete endogenous complement) taken from naive mice or mice given adjuvant alone supported complement-dependent bactericidal activity against strain FA1090 when normal human serum (NHS) was added as the source of complement; FA1090 is highly resistant to the bactericidal action of NHS alone (19). Control mouse sera plus heat-inactivated (56°C for 30 min) NHS failed to kill gonococci, suggesting that observed killing was complement dependent. A systematic analysis of the bactericidal activity of heat-inactivated sera with added NHS from several strains of naive mice was carried out (see Table S3 in the supplemental material). Except for *Rag^−/−^* and JhD mice, which both lack antibody, heat-inactivated sera from all other mice tested supported human complement-dependent killing of N. gonorrhoeae. Similarly, bactericidal activity was supported by sera from CD1 mice housed at the Children’s Hospital Oakland Research Institute, Oakland, CA, given alum adjuvant (20).

10.1128/mBio.02552-19.8 TABLE S3 Bactericidal activity of naïve mouse sera against N. gonorrhoeae FA1090. Download Table S3, PDF file, 0.04 MB. Copyright © 2019 Gulati et al. 2019 Gulati et al. This content is distributed under the terms of the Creative Commons Attribution 4.0 International license.

Absorption of naive or adjuvant control mouse sera against anti-mouse IgM-linked agarose, but not protein A/G agarose (depletes IgG), abrogated bactericidal activity (survival of >80%), revealing IgM as the antibody subclass responsible for the underlying bactericidal activity. Mouse sera used in (human) complement-dependent bactericidal assays in antigen optimization and adjuvant selection studies were therefore depleted of IgM (Fig. S2). We measured anti-LOS IgG levels in 5 immune sera before and after passage through anti-mouse IgM agarose and noted that absorption resulted in a mean decrease in IgG concentration of 13.6% (range, 9.2 to 18.5%).

Serum bactericidal assays with IgM-depleted immune and adjuvant control sera performed using normal human serum (NHS) as the source of complement (Fig. S2B) showed that killing of gonococci mirrored anti-LOS IgG titers. Based on these data, GLA-SE was chosen as the lead adjuvant because it has received approval for human use (21, 22).

### Immunogenicity and bactericidal activity of antisera from mice immunized with TMCP2 and GLA-SE adjuvant.

**FIG 2:**
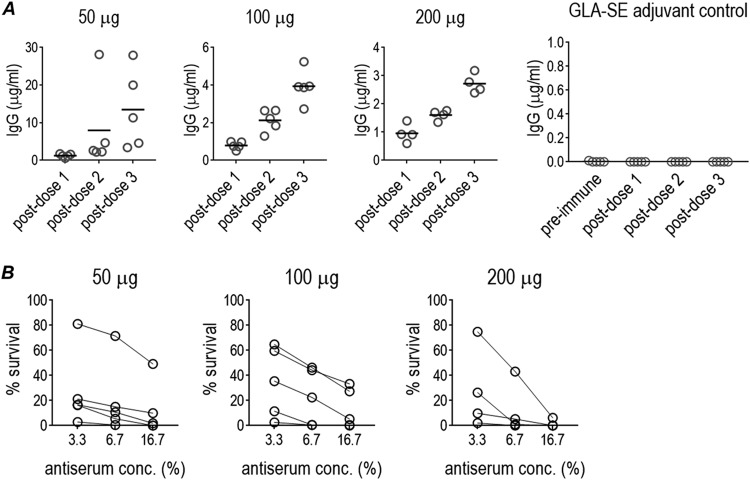
Immunogenicity (A) of TMCP2/GLA-SE and functional activity of elicited antibodies (B). Six-week-old female BALB/c mice were immunized with three doses of TMCP2 given at 50, 100, or 200 μg per dose with GLA-SE adjuvant (5 μg) every 3 weeks or with GLA-SE alone (adjuvant control). The groups immunized with 50 or 100 μg of TMCP2 per dose contained 5 mice each; the group given 200 μg/dose of TMCP2 had 4 mice. (A) Antibody levels against LOS purified from N. gonorrhoeae strain 15253 (elaborates the 2C7 epitope optimally). Anti-LOS IgG levels against 15253 LOS were measured in sera obtained 2 weeks after each dose. Note the different *y* axes in the three graphs. None of the sera showed detectable IgG antibody binding to 15253 Δ*lgtG* LOS. GLA-SE alone (adjuvant control) showed no detectable anti-gonococcal LOS IgG. (B) Bactericidal activity of immune sera against N. gonorrhoeae FA1090. Post-dose 3 sera were tested for their ability to kill N. gonorrhoeae FA1090 using human complement (16.7% [vol/vol]) as the complement source. The concentrations of heat-inactivated mouse antiserum in the reaction mixtures are shown on the *x* axes. All antisera from mice immunized with GLA-SE alone did not show any bactericidal activity (>100% survival). (Table S4A shows numerical data.)

An initial immunization experiment utilized inoculation plus boosting at 3 and 6 weeks (3 doses) of 5 mice each with 50, 100, or 200 μg (4 mice) of TMCP2 combined with GLA-SE adjuvant. Immunization elicited anti-LOS IgG responses, measured 2 weeks after final boosting, that exceeded 2 μg/ml in every mouse (Fig. 2A). Post-dose 3 responses in 3 of 5 mice given the 50-μg dose exceeded 10 μg/ml. Mice immunized with GLA-SE alone did not elicit IgG responses against LOS. Specificity was demonstrated by nonreactivity of immune sera with 15253 Δ*lgtG* LOS (data not shown).

**FIG 3:**
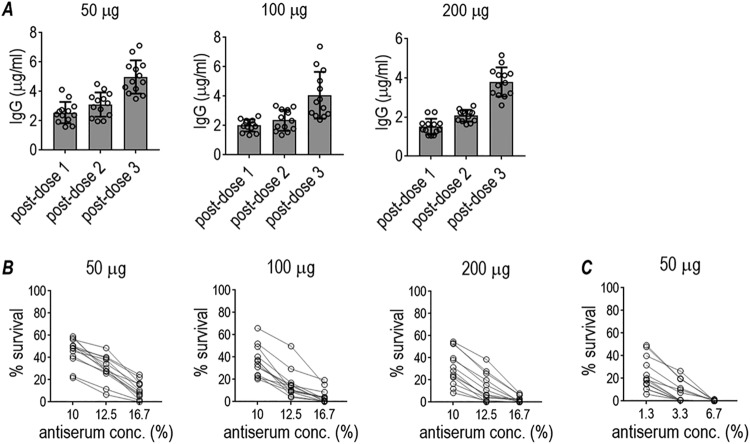
Immunogenicity and functional activity of antibodies elicited in groups of mice immunized with TMCP2/GLA-SE to evaluate broader functional efficacy. Six-week-old female BALB/c mice were immunized with TMCP2 given at 50, 100, or 200 μg per dose with GLA-SE adjuvant (5 μg) every 3 weeks, or with GLA-SE alone (adjuvant control; not shown). Each group contained 13 mice and represented mice not used for challenge studies in Fig. 4. (A) Anti-LOS antibody levels in immune sera. Sera obtained 2 weeks after each dose were assayed for antibody levels against N. gonorrhoeae 15253 LOS. Note the different *y* axes in the three graphs. None of the antisera from mice immunized with GLA-SE alone (adjuvant control) showed measurable anti-gonococcal LOS IgG levels. (B) Bactericidal activity of immune sera against strain FA1090. Post-dose 3 sera used at concentrations (indicated on the *x* axis) of 10% (1/10 dilution), 12.5% (1/8 dilution), or 16.7% (1/6 dilution) were tested for their ability to kill N. gonorrhoeae FA1090 using NHS (20% [vol/vol]) as the complement source. The *y* axis shows the percentage of bacterial survival at 30 min relative to 0 min. (Table S4B shows the numerical data.) (C) Bactericidal activity against strain MS11 of immune sera from mice immunized with three doses of 50 μg, each given 3 weeks apart. Bactericidal assays were performed as described in panel B, except that immune sera were tested at (lower) concentrations: 1.3, 3.3, and 6.7% with 6.7% human complement (IgG/IgM-depleted NHS [Pel-Freez]) added as a source of complement. None of the IgM-depleted antisera from adjuvant control mice (given GLA-SE alone) showed any bactericidal activity (>100% survival). (Table S4C shows the numerical data.)

10.1128/mBio.02552-19.9 TABLE S4 (A) Complement-dependent killing of N. gonorrhoeae FA1090 in intact and IgM-depleted immune sera (data displayed graphically in Fig. 2B). (B) Complement-dependent killing of N. gonorrhoeae FA1090 in intact and IgM-depleted immune sera. (Data are displayed graphically in Fig. 3B.) (C) Complement-dependent killing of N. gonorrhoeae MS11 in intact and IgM-depleted immune sera. (Data are displayed graphically in Fig. 3C.) (D) Complement-dependent killing of N. gonorrhoeae FA1090 in intact and IgM-depleted immune sera. (Data are shown graphically in Fig. 5C.) Download Table S4, PDF file, 0.1 MB. Copyright © 2019 Gulati et al. 2019 Gulati et al. This content is distributed under the terms of the Creative Commons Attribution 4.0 International license.

Curiously, the “natural” bactericidal IgM antibodies noted in naive mice or mice given adjuvants alone seen in the antigen optimization and adjuvant selection studies were not observed in subsequent experiments (performed after June 2018). Intact immune sera taken 2 weeks after the last immunization were tested for bactericidal activity against strain FA1090 (Fig. 2B; numerical data are shown in Table S4A in the supplemental material). All immune sera showed >50% killing when tested at a final dilution of 1:6 (immune serum concentration of 16.7%). Mice that received GLA-SE alone did not show IgG responses against LOS and did not support complement-dependent bactericidal activity of FA1090 (Table S4A). Depletion of IgM in these sera did not reduce bactericidal activity significantly, indicating that IgG was responsible for the bactericidal activity in these immune sera (Table S4A).

### *In vitro* and *in vivo* efficacy of TMCP2 tested at different doses.

In a second, larger experiment, three groups of female BALB/c mice (*n* = 25 per group) were immunized using the same protocol used above; a fourth group (*n* = 25) received GLA-SE alone. Two weeks after the third immunization, mice in the diestrus phase of the estrous cycle were treated with Premarin and challenged with N. gonorrhoeae strain FA1090 (*n* = 6/group) or MS11 (*n* = 6/group). Sera from mice not used for challenge (*n* = 13 in each group) were collected by cardiac bleeding for immunologic studies (IgG LOS ELISA and serum bactericidal assays).

Anti-LOS IgG levels were measured in immune sera (*n* = 13 in each group) from mice not used for challenge experiments. As shown in Fig. 3A, anti-LOS IgG levels in intact immune sera exceeded 2 μg/ml in all mice. The mean anti-LOS IgG levels in the 50-, 100-, and 200-μg/dose groups were 4.98, 4.06, and 3.8 μg/ml, respectively. As was shown in the experiment using a smaller number of mice (Fig. 2A), none of the sera from mice immunized with GLA-SE alone (adjuvant control) showed detectable antigonococcal LOS IgG levels (not shown). We also measured anti-LOS IgM levels in pooled immune and pooled adjuvant sera (Fig. S3A, i). Similar IgM binding was measured to 15253 LOS (2C7 positive) and 15253 Δ*lgtG* LOS (2C7 negative) in immune sera and adjuvant sera, indicating no apparent IgM response to the 2C7 epitope in mice (Fig. S3A, i). Predictably, passage of sera over anti-mouse IgM agarose abrogated IgM detection in both immune and adjuvant control sera. We also compared IgG binding to LOS in the same sera (Fig. S3A, ii); only immune serum, but not adjuvant control serum, bound 15253 LOS; no IgG binding to 15253 Δ*lgtG* LOS was detected.

10.1128/mBio.02552-19.4 FIG S3 (A) Reactivity of IgM in immune and adjuvant control sera against LOS and FA1090. (B) Specificity of IgG response to the 2C7 LOS epitope. Post-dose 3 sera from adjuvant (GLA-SE) control mice (experiment described in Fig. 5) that were not infected with N. gonorrhoeae were tested for IgG reactivity with 2C7-positive LOS (left graph), and sera from TMCP2/GLA-SE-immunized mice not used for gonococcal challenge were tested for reactivity against 2C7-negative LOS (right graph). Download FIG S3, PDF file, 0.2 MB. Copyright © 2019 Gulati et al. 2019 Gulati et al. This content is distributed under the terms of the Creative Commons Attribution 4.0 International license.

10.1128/mBio.02552-19.10 TABLE S5 Serum and vaginal anti-LOS antibody levels in BALB/c mice immunized with TMCP2/GLASE. Download Table S5, PDF file, 0.03 MB. Copyright © 2019 Gulati et al. 2019 Gulati et al. This content is distributed under the terms of the Creative Commons Attribution 4.0 International license.

The 13 individual immune sera in each group collected 2 weeks after the 3rd dose of TMCP2 had been administered were tested for ability to kill FA1090 and MS11 in complement-dependent serum bactericidal assays. Dose-dependent killing of N. gonorrhoeae was seen in all three groups. Notably, all sera, when tested at final concentrations of 16.7% (1/6 dilution), resulted in >50% complement-dependent killing (<50% survival) of FA1090 (Fig. 3B). Strain MS11 was more susceptible than FA1090 to complement-dependent killing by sera from mice that had been immunized with the 50-μg/dose schedule. All 13 immune sera, when used at final concentrations of 3.3%, killed MS11 at >60%; 6.7% immune serum concentrations resulted in >98% killing (Fig. 3C). Consistent with the observation that post-dose 3 sera did not possess specific IgM against the 2C7 epitope (Fig. S3A), we did not note significant decreases in killing of either FA1090 (Table S4B) or MS11 (Table S4C) when IgM was depleted. None of the antisera from adjuvant control (GLA-SE alone) mice showed bactericidal activity (>100% survival) (Table S4B and Table S4C).

**FIG 4:**
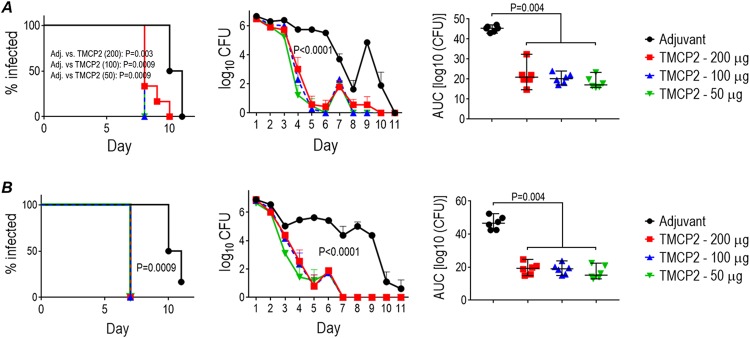
Efficacy of TMCP2/GLA-SE against N. gonorrhoeae strains FA1090 and MS11 in the mouse vaginal colonization model. Six-week-old female BALB/c mice were immunized with TMCP2 (either 50, 100, or 200 μg/dose) plus GLA-SE (5 μg) or with GLA-SE alone (adjuvant control) at 0, 3, and 6 weeks. Quantitation of antibody concentrations against 15253 LOS and serum bactericidal antibody titers in corresponding groups of mice immunized at the same time are shown in Fig. 3. Two weeks after the last immunization, 12 mice in each group in the diestrus phase of the estrous cycle were treated with Premarin; six mice in each of the four groups were infected intravaginally with strain FA1090 (5 × 10^7^ CFU; *n* = 6) or with strain MS11 (4.2 × 10^7^ CFU) on day 0. Efficacy of TMCP2/GLA-SE against FA1090 and MS11 is shown in panels A and B, respectively. Sera from 13 simultaneously immunized mice in each group were used for immunogenicity studies (Fig. 4). The graphs on the left (Kaplan-Meier analysis) show time to clearance of infection. Groups were compared using the Mantel-Cox log-rank test. Significance was set at 0.008 (Bonferroni’s correction for 4 groups). The middle graphs show log_10_ CFU versus time. Graphs on the right show bacterial burdens consolidated over time (area under the concentration-time curve [log_10_ CFU] analysis). The median and 95% confidence intervals are shown for each group. The four groups were compared using the nonparametric Kruskal-Wallis equality-of-populations rank test. The χ^2^ test results with ties (3 df) were 14.36 (*P* = 0.0025) for FA1090 (A) and 13.69 (*P* = 0.0034) for MS11 (B). Pairwise comparisons across groups (indicated with the graphs) were made with the two-sample Wilcoxon rank-sum test and applying the Kruskal-Wallis equality-of-populations rank test for comparisons across more than two groups.

As shown in Fig. 4 (panels A and B show experimental challenge data with FA1090 and MS11, respectively), all three doses of TMCP2 accelerated clearance and diminished colonization with each of the gonococcal strains over the 11-day course, compared to the adjuvant control. The rate of decline of CFU with time (middle graphs) and the area under the concentration-time curve analyses (right graphs) showed similar vaccine efficacy at all three doses and against both isolates.

### Efficacy of TMCP2 using a biweekly schedule.

**FIG 5:**
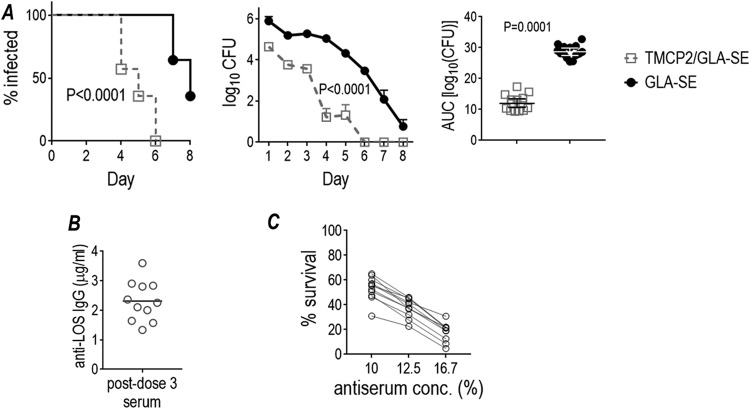
Efficacy of TMCP2/GLA-SE against N. gonorrhoeae FA1090 in the mouse vaginal colonization model using a biweekly 3-dose schedule. Six-week-old female BALB/c mice (*n* = 25/group) were immunized with 50 μg/dose TMCP2 plus GLA-SE (5 μg) or with GLA-SE alone (adjuvant control) at 0, 2, and 4 weeks. Two weeks after the last immunization, 14 mice in each group in the diestrus phase of the estrous cycle were treated with Premarin and infected intravaginally with strain FA1090 (10^7^ CFU) on day 0. Sera from the remaining 11 mice in each group was collected by cardiac puncture for use in immunologic studies. (A) The graph on the left shows time to clearance of infection (Kaplan-Meier analysis; groups were compared using the Mantel-Cox log-rank test). The graph in the middle shows log_10_ CFU versus time. The median and 95% confidence intervals are shown for each group. The graph to the right shows bacterial burdens consolidated over time (area under the concentration-time curve [log_10_ CFU] analysis). Pairwise comparisons between groups were made with the two-sample Wilcoxon rank-sum (Mann-Whitney) test. (B) Anti-LOS antibody levels in immune sera. Sera obtained 2 weeks after the 3rd vaccine dose were assayed for antibody levels against N. gonorrhoeae 15253 LOS. None of the antisera from mice immunized with GLA-SE alone (adjuvant control) showed measurable anti-gonococcal LOS IgG levels, and none of the immune sera reacted with 2C7-negative LOS (Fig. S3B). (C) Bactericidal activity of immune sera. Sera obtained 2 weeks after the 3rd vaccine dose used at concentrations of 10% (1/10 dilution), 12.5% (1/8 dilution) or 16.7% (1/6 dilution) (concentrations indicated on the X-axis) were tested for their ability to kill N. gonorrhoeae FA1090 using human complement (16.7% [vol/vol]) as the complement source. *y* axis, % bacterial survival at 30 min relative to 0 min. None of the IgM-depleted antisera from adjuvant control mice (given GLA-SE alone) showed any bactericidal activity (>100% survival [Table S4D]).

We also examined a compressed schedule of immunization (dosing at 2-week instead of 3-week intervals). Mice were immunized with 50 μg of TMCP2 plus GLA-SE using three 50-μg doses, each given 2 weeks apart. Two weeks after the third dose, mice (*n* = 14/group) were challenged with strain FA1090 (10^7^ CFU). This compressed immunization regimen also effectively attenuated gonococcal colonization (Fig. 5A) compared to GLA-SE adjuvant alone. The mean anti-LOS level in sera from 11 fully immunized mice was 2.31 μg/ml (standard deviation, 0.68; range 1.33 to 3.59 μg/ml) (Fig. 5B). Specificity of the immune responses were shown by nonreactivity of adjuvant control sera with 2C7-positive LOS and nonreactivity of immune sera against 2C7-negative LOS purified from 15253 Δ*lgtG* (Fig. S3B). All sera showed bactericidal activity (>50% killing at 1:6 dilution [16.7% antiserum concentration]) (Fig. 5C), similar to that achieved by similar or higher TMCP2 dosing administered at longer intervals (Fig. 4). IgM depletion from sera did not change bactericidal activity significantly (Table S4D).

In a separate experiment, we immunized 5 mice with TMCP2 (50 μg/dose) plus GLA-SE, or GLA-SE alone at 0, 2, and 4 weeks and collected serum and vaginal swabs 2 weeks after the third dose for measurement of anti-LOS IgG. Anti-LOS IgG was detected in vaginal swabs of the 5 mice given TMCP2 (see Table S5 in the supplemental material).

## DISCUSSION

In this study, we show that a candidate gonococcal peptide vaccine that elicits bactericidal antibodies against N. gonorrhoeae significantly reduces the duration and burden of gonococcal cervicovaginal colonization in BALB/c mice. Combining TMCP2 with GLA-SE, which elicits a Th1-biased response (22–25), evoked 2C7 epitope-specific IgG antibody responses after immunization with three intramuscular doses. Our prior studies using the octameric peptide forerunner of TMCP2 (13) and other studies in mice (28) suggest that a Th1 response clears infection and induces a memory response, which may have contributed to the efficacy of GLA-SE.

A limitation of the octameric peptide vaccine used in our previous study (13) was heterogeneity of the molecular mass of the preparation, likely the result of formation of inter- and intramolecular disulfide bonds. To circumvent this problem, we devised a novel synthetic method in which each peptide subunit was first circularized with a covalent, nonreducible thioether bond; each of the circularized subunits was then linked to a dendrimeric Glu backbone. The resulting tetrapeptide, TMCP2, showed a single peak on mass spectroscopy. This relatively facile synthesis process yielded a compound of greater than 95% purity, which is suitable for scale-up and production of an inexpensive gonococcal vaccine candidate.

Activation of the classical pathway of complement requires engagement of the C1 complex (subunits of C1q, C1r and C1s), triggered by Fc antibody domains. Upon binding to surfaces, Fc domains of proximate IgG molecules form ordered hexamers through noncovalent interactions (27), which then simultaneously engage multiple globular heads of C1q, also a hexameric molecule. Multimeric interactions between globular domains of C1q and Fc convert otherwise low-affinity monomeric IgG Fc-C1q associations to interactions of high avidity, which permits autocatalysis of C1r and further complement activation. An effective bactericidal antibody requires a critical density of surface targets to engage C1q and permit complement activation. On a molar basis, LOS is the most abundant gonococcal outer membrane molecule and serves as a convenient target for binding of closely spaced antibody molecules whose Fc domains can then readily engage the C1 complex and activate complement. We have shown that an intact complement system is necessary and sufficient for efficacy of passively administered bactericidal MAb 2C7—given either systemically or intravaginally—in the mouse vaginal colonization model (18). The importance of complement in host defenses against gonorrhea is highlighted by the observation that both congenital and acquired defects of individual terminal components of complement are associated with an increased incidence of disseminated gonococcal infection (DGI) (28–31). Based on our data with MAb 2C7, we propose that attenuation of colonization by antibodies elicited by TMCP2 vaccine also occurs via complement-dependent bactericidal antibody activity. A vaccine candidate developed by Liu and colleagues that comprises gonococcal outer membrane vesicles plus microencapsulated interleukin-12 (IL-12) given intravaginally requires B cell activity, presumably to produce antibodies (32); however, a requirement for complement has not been shown.

Similar to our findings, no other gonococcal vaccine candidates tested in the cervicovaginal colonization model in estradiol-treated mice show sterilizing immunity (26, 32, 33). Immunomodulatory effects of estrogen (34) may curb immune defenses that are important to clear N. gonorrhoeae. Studies over 50 years ago showed that the activity of terminal complement components in male mice exceeds, by 8- to10-fold, activity in female mice (35). Administration of testosterone increases complement activity; estrogen has the opposite effect (36). Therefore, a vaccine antibody response that requires terminal complement for its efficacy may simultaneously require waning of the suppressive effects of estrogen on synthesis of terminal complement components before full bactericidal activity is restored.

A retrospective epidemiologic analysis showed that a detergent-extracted meningococcal outer membrane vesicle (dOMV) vaccine called MeNZB designed and implemented in a widespread vaccination program to control an epidemic of group B meningococcal disease in New Zealand, showed diminished coverage in populations subsequently infected with N. gonorrhoeae—calculated as 31% effectiveness of MenZB in decreasing gonococcal infection—reduced to 14% in populations coinfected with N. gonorrhoeae and chlamydia, a frequent clinical occurrence (37–39). Meningococci and gonococci share several similarities, and it is possible that one or more proteins in MeNZB that cross-react with N. gonorrhoeae may elicit protective immune responses. Individuals administered a licensed group B meningococcal vaccine, Bexsero, which contains 5 recombinant meningococcal protein antigens in addition to the same dOMV that constitutes MeNZB (40), elicit antibodies, immunochemically, that cross-react with N. gonorrhoeae (41). Antibodies elicited by immunizing mice with Bexsero or MeNZB were reported to support bactericidal activity against N. gonorrhoeae (42). However, a separate study did not reveal bactericidal antibody activity against N. gonorrhoeae FA1090 in immune sera from individuals vaccinated with Bexsero (20). How MeNZB provides protection against gonorrhea remains unclear; possible additional mechanisms may include reduction of adhesion of N. gonorrhoeae to human cervical cells (42). Currently, several gonococcal vaccine candidates are undergoing preclinical evaluation (reviewed in reference 5), some of which elicit bactericidal activity (43–45).

Three glycosyltransferase genes (*lgtA*, *lgtC*, *lgtD* involved in LOS biosynthesis are phase-variable because of slipped-strand mispairing of homopolymeric poly(G) tracts in their open reading frames (46, 47); a fourth, *lgtG*, containing a poly(C) tract, is also phase variable (12, 46, 48–50). Expression of the 2C7 epitope requires *lgtG* to be phase varied “on”; the expression status of the three other *lgt* genes modulates binding of MAb 2C7 to LOS (11). As discussed above, the 2C7 LOS epitope is expressed almost universally *in vivo* and by minimally passaged isolates. The 2C7 epitope was identified in 64 of 68 (94%) gonococcal isolates examined directly in cervical secretions from women in a sexually transmitted disease clinic in Boston in the 1990s and 96 of 101 (95%) of randomly chosen minimally (second-passage) isolates (7). Recently, we reported that 100% of 75 minimally passaged isolates from China also expressed the 2C7 epitope (15). Importantly, MAb 2C7 was bactericidal (>50% killing) against each of 62 isolates tested in complement-dependent bactericidal assays, including strains that expressed low levels of the epitope (15). Because LOS is the most abundant outer membrane molecule on gonococci, expression of the 2C7 epitope on even a small fraction of LOS may permit binding of antibody at a density sufficient to engage the C1 complex and activate the classical pathway. We speculate that widespread expression of this epitope results from the gonococcal ability to sialylate lactose expressed from HepII, which facilitates engagement of Siglec receptors (16). Many Siglec receptors signal through their immunoreceptor tyrosine-based inhibitory motif (ITIM) tails (51) to dampen host inflammatory responses that otherwise sense invading pathogens (52, 53). Neu5Ac (sialic acid) that caps lactose from HepII also inhibits complement C3 deposition on the bacterial surface (15). Genetic deletion of *lgtG* or *lst* (sialyltransferase) from a strain whose only site for sialylation is the terminal Gal of HepII lactose markedly attenuates the ability of N. gonorrhoeae to colonize the vagina of estradiol-treated mice (13, 15). Additional evidence for the importance of *lgtG* expression was provided by Lam and Gray-Owen, who showed that serial passage of N. gonorrhoeae in mice was accompanied by increased fitness of bacteria with each generation—i.e., an increasing fraction of mice could be infected with bacteria recovered from each successive mouse passage. Intriguingly, there was a reproducible positive selection for gonococcal variants with *lgtG* “on” (54). Resistance to antibodies elicited by a “2C7 vaccine” would require *lgtG* to be turned completely “off.” Based on the accumulated evidence from studies of minimally passaged isolates, bacteria examined directly *ex vivo* from humans (without passage on media), and studies in mice, as discussed above, we propose that mutations in gonococci that eliminate 2C7 LOS epitope would render the organism less fit and avirulent. From a public health perspective, translation to a decrease in burden and duration of infection can have profound effects on disease pathology and transmission. In conclusion, TMCP2 represents an important step forward in the development of a safe, economical, and effective gonococcal vaccine or subcomponent thereof.

## MATERIALS AND METHODS

### Bacterial strains.

N. gonorrhoeae strains FA1090 (Opa^+^ Pil^+^) (55), MS11 (Opa^+^ Pil^+^) (56), 15253 (57), and 15253 Δ*lgtG* (isogenic mutant of 15253 with *lgtG* deleted, which does not react with MAb 2C7) (15) have been described previously.

### Purification of LOS.

LOS was purified from strains 15253 and 15253 Δ*lgtG* using the hot water phenol extraction method (58).

### Materials for production of TMCP2.

The 4 branched reverse MAP (multiple antigenic peptide) was synthesized using a solid-phase approach for the thioether peptide component and a classical solution phase method for production of the reverse MAP core and final tetra MAP construct. Briefly, the thioether monomer was synthesized on an Fmoc-Lys-Wang resin using standard Fmoc-tBu amino acid derivatives. At the completion of assembly of the protected linear peptide sequence: H-Gly-Pro-(Ile-Pro-Val-Leu-Asp-Glu-Asn-Gly-Leu-Phe-Ala-Pro)-Gly-Pro-Cys-Lys-OH, bromoacetic acid was coupled to the N terminus via symmetric anhydride coupling. The peptide Br-Ac-Gly-Pro-Ile-Pro-Val-Leu-Asp-Glu-Asn-Gly-Leu-Phe-Ala-Pro-Gly-Pro-Cys-Lys-OH was cleaved from the solid support and simultaneously deprotected using trifluoroacetic acid containing H_2_O and triisopropyl silane as cationic scavengers. The crude linear peptide was purified using preparative HPLC and subsequently cyclized by dilution into 1% Na_2_CO_3_ buffer to a concentration of 0.3 mg/ml. Cyclization was completed at 18 h, and the cyclic thioether peptide (structure indicated as “MAP Core Peptide 2” in Fig. 1A) was purified via preparative RP-HPLC. Fractions that had a purity of >90% by analytical HPLC were pooled and lyophilized. The cyclic thioether monomer peptide was characterized using ESI-MS (calculated *m/z* = 1,864.17; measured *m/z* = 1,864.88).

The reverse MAP core construct was synthesized starting with Ac-β-Ala-OH. H-Glu(OBzl)_2_ was coupled to the Ac-β-Ala-OH using diphenylphosphoryl azide. The benzyl groups were liberated via catalytic hydrogenation. Ac-β-Ala-Glu-OH was reacted subsequently with 2 equivalents of H-Glu(OBzl)_2_. Benzyl groups were removed again via catalytic hydrogenation. The final reverse tetra-MAP core peptide Ac-β-Ala-Glu[Glu-OH]_2_ (“Reverse MAP Core” in Fig. 1A) was isolated and characterized by ESI MS: calculated *m/z* = 519.19 and measured *m/z* = 519.22. This compound was subsequently activated to the tetra NHS ester in the presence of diisopropyl carbodiimide.

The tetra-MAP construct TCMP2 was synthesized by adding 4.2 equivalents of the cyclic thioether MAP core peptide 2 to the reverse MAP core: Ac-β-Ala-Glu[Glu(OSu)_2_]_2_. Following coupling for 20 h, the crude peptide tetra-MAP TMCP2 was isolated by preparative RP-HPLC. The resulting fractions with a purity of >95% were pooled and lyophilized. The final product, TCMP2, was isolated and characterized by ESI MS. The structure for TMCP2 is shown in Fig. 1A. Figure S4 shows a summary of the synthesis procedure.

10.1128/mBio.02552-19.5 FIG S4 Schematic summarizing the steps in the synthesis of TMCP2. Download FIG S4, PDF file, 0.2 MB. Copyright © 2019 Gulati et al. 2019 Gulati et al. This content is distributed under the terms of the Creative Commons Attribution 4.0 International license.

### Immunization of mice.

Six-week-old female BALB/c mice were immunized with TMCP2 (dose specified for each experiment) and GLA-SE (5 μg) as the adjuvant. Control mice received GLA-SE adjuvant alone. Sera were collected 2 weeks after each immunization.

### ELISA to measure anti-LOS antibody levels.

Microtiter wells were coated with LOS purified from 15253 or 15253 Δ*lgtG* (80 μg/ml) in phosphate-buffered saline (PBS) as described previously (13). Serial dilutions of immune sera were dispensed into wells, and bound anti-LOS antibody was disclosed with anti-mouse IgG conjugated to alkaline phosphatase. A standard curve for mouse IgG was generated by coating wells with anti-mouse IgG (Sigma) and pure mouse IgG (Sigma), also as described previously (13). Mouse IgM bound to LOS was detected with anti-mouse IgM conjugated with alkaline phosphatase (Sigma).

### Inhibition ELISA.

In order to compare antigenicity of MAP core peptide 2 (monomeric cyclic CP2) and composite TMCP2 compounds with LOS that expresses the 2C7 epitope, we performed inhibition ELISA using mouse MAb 2C7 (7, 17) reacted with the individual compounds to assess inhibition of binding (residual binding) of MAb to LOS by each compound. Microtiter wells were coated with LOS (80 μg/ml in PBS) purified from N. gonorrhoeae strain 15253. Wells were washed and blocked with PBS–0.05% Tween 20. MAb 2C7 (0.04 μg/ml), either alone or containing monomeric cyclic peptide CP2 (1.07 to 1070 μM) or composite TMCP2 (at concentrations ranging from 0.25 to 250 μM), was dispensed into LOS-coated wells. Residual binding of 2C7 MAb was measured with anti-mouse IgG conjugated to alkaline phosphatase.

Bound IgG was quantitated using a mouse IgG standard curve (17), and the percentage of inhibition was determined compared to reaction mixtures that contained LOS (full inhibition).

### Depletion of mouse IgM.

Because earlier we had observed that sera from naive and mice given adjuvant alone manifest bactericidal activity mediated by IgM (see Results), we depleted mouse IgM from sera collected as part of antigen optimization and adjuvant selection studies (described above). Sera from mice were mixed with anti-mouse IgM-agarose (Sigma; binding capacity, ≥0.4 mg/ml) (1 volume of packed agarose to 1 volume of serum diluted 1:1) in a 2.0-ml microspin filter column (Costar) with a pore size of 0.22 μm for 15 min at 22°C. Filter-sterilized IgM-depleted sera were collected by centrifugation of the mixture at 1,000 × *g* for 2 min and heat inactivated (56°C for 30 min) to eliminate intrinsic complement activity in mouse serum prior to use in serum bactericidal assays where human complement was substituted.

### Serum bactericidal assays.

Serum bactericidal assays were performed as described previously (59). Bacteria that had been harvested from an overnight culture on chocolate agar plates were repassaged onto fresh chocolate agar and allowed to grow for 6 h at 37°C in an atmosphere containing 5% CO_2_. Bacteria were then suspended in Hanks’ balanced salt solution (HBSS) containing 1 mM MgCl_2_ and 0.15 mM CaCl_2_ (HBSS^++^) for use in serum bactericidal assays. About 2,000 CFU was incubated with serial dilutions of immune mouse sera (heat-inactivated and IgM depleted) in the presence or absence of 20% normal human serum (NHS) as a source of human complement. Serum bactericidal assays with strain MS11 were performed with IgG- and IgM-depleted NHS (human complement; Pel-Freez) because MS11 is susceptible to killing by NHS. The final volumes of bactericidal reaction mixtures were 150 μl. Aliquots of 25-μl reaction mixtures were plated onto chocolate agar in duplicate at the beginning of the assay (time zero [*t*_0_]) and after incubation at 37°C for 30 min (*t*_30_). Survival was calculated as the number of viable colonies at *t*_30_ relative to *t*_0_.

### Mouse protection experiments.

Use of animals in this study was performed in strict accordance with the recommendations in the *Guide for the Care and Use of Laboratory Animals* of the National Institutes of Health (60). The protocol was approved by the Institutional Animal Care and Use Committee (IACUC) at the University of Massachusetts Medical School. The BALB/c mouse model of vaginal colonization described by Jerse was used (61). Two weeks after the last immunization, mice in the diestrus phase of the estrous cycle were started on treatment (that day) with 0.1 mg Premarin (Pfizer) in 200 μl of water, given subcutaneously on each of 3 days: days −2, 0, and +2 (before, the day of, and after gonococcal inoculation) to prolong the estrus phase of the reproductive cycle and promote susceptibility to N. gonorrhoeae infection. Antibiotics (vancomycin and streptomycin) ineffective against N. gonorrhoeae were also used to reduce competitive microflora (62). Mice were infected on day 0 with either strain FA1090 or MS11 (inoculum specified for each experiment). Vaginas were swabbed daily to enumerate gonococcal CFU.

### Statistical analyses.

Experiments that compared clearance of N. gonorrhoeae in independent groups of mice estimated and tested three characteristics of the data (13): time to clearance; longitudinal trends in mean log_10_ CFU and the cumulative CFU as area under the concentration-time curve (AUC). Statistical analyses were performed using mice that initially yielded bacterial colonies on day 1 and/or 2. Median time to clearance was estimated using Kaplan-Meier survival curves; times to clearance were compared between groups using the Mantel-Cox log-rank test. Mean log_10_ CFU trends over time were compared between groups using a linear mixed model with mouse as the random effect using both a random intercept and a random slope. A quadratic or cubic function in time was determined to provide the best fit; random slopes were linear in time. A likelihood ratio test was used to compare nested models (with and without the interaction term of group and time) to test whether the trend differed over time between the two groups. The mean AUC (log_10_ CFU versus time) was computed for each mouse to estimate the bacterial burden over time (cumulative infection); the means under the curves were compared between groups using the nonparametric two-sample Wilcoxon rank-sum (Mann-Whitney) test because distributions were skewed or kurtotic. The Kruskal-Wallis equality-of-populations rank test was also applied to compare more than two groups in an experiment.
