# Supplementary material for: Preclinical Efficacy of a Lipooligosaccharide Peptide Mimic Candidate Gonococcal Vaccine
Source: mBio. 2019 Nov 5;10(6):e02552-19. doi: 10.1128/mBio.02552-19 (PMC6831779; doi:10.1128/mBio.02552-19)
Supplement: TEXT S1 [file mBio.02552-19-s0001.pdf]

Supplemental Text

Peptide constructs prior to derivation of the lead candidate (numbers in brackets refer to compounds listed in Table S2 that indicate chemical structures screened for use in this study)

The Tetra-MAP preparation (termed Tetra MAP 1 **[1]**; and likewise, an Octa-MAP preparation (termed Octa MAP1 **[2]**) that had been used in our previous immunization/ challenge study (challenge performed with Octa-MAP 1 only) (Gulati S, Zheng B, Reed GW, Su X, Cox AD, St Michael F, Stupak J, Lewis LA, Ram S, Rice PA. 2013. PLoS Pathog 9:e1003559) was synthesized by 'step-wise' addition of amino acids to a poly-lysine core. This resulted in a heterogenous preparation (**Table S1a;** western blot showed for the Octa-MAP preparation only) that while effective in immunization/ challenge studies, was not suitable for further clinical development.

To overcome heterogeneity of the immunogen, all further attempts used a 'modular' synthesis approach, where highly purified (and cyclized where indicated) peptides were added 'en bloc' to the core. Synthesis of Tetra-MAP (termed Tetra MAP 1.1 **[3]**) using the 'modular' approach and the original core yielded the same compound as the 'step-wise' prepared material. A 2nd compound that substituted serine for Cys at the N-terminal Cys residue (termed Tetra MAP 1.2 **[4]**) to reduce internal disulfide formation was also produced. Neither compound was superior to 'step-wise' produced material in immunogenicity studies (shown below in **Table S1g**).

Construction of a Tetra-MAP was attempted utilizing 4 maleimide (MAL) residues from a Lys dendrimer core (**Table S1b**) that would link sulfydryl groups (-SH) of the N-terminal cysteine directly to the (poly) lysine core. However, additional reaction

of the maleimide residues with primary amines in the monomeric peptide in this reaction resulted in a product that was poorly soluble and highly heterogenous; this approach was also abandoned.

In experiments to modify linkages of the monomeric peptide mimitope (PEP1) to the core, an Octa-MAP was made by first protecting the N-terminal Cys of the mimitope (PEP1) with a tert-butylsulfinyl (StBu) group **[5]** or replacing the N-terminal Cys with serine **[6]** and then linking the C-terminal Cys of the monomeric peptide to a heterobifunctional cross-linker intermediary molecule (MAL-PEG_4-NHS), through the maleimide (MAL) reactive group (**Table S1c**). The reactive N-hydrosuccinimide (NHS) at the N-terminus was displaced and PEP1-MAL-PEG_4 molecules were amide-linked to each of 8 Lys residues of a Poly-Lys backbone. Polyethylene glycol (PEG_4) was included to ensure a water-soluble product. Unfortunately, this synthetic process, although yielding a homogenous compound (**Table S1d**), was too inefficient and expensive for production and scale-up.

In order to eliminate heterogeneity, three processes were considered to modify the monomeric peptides: (i) elimination or replacement of terminal cysteines, (ii) use of Click chemistry as means to link peptide to the core and (iii) use of of Reverse MAP synthesis (Reverse MAP entails positioning carboxyl groups in the core for modification by amine components from the peptide monomers) to link several versions of the initial peptides in stable cyclic forms using either disulfide or thioether bonds resulting in stable cyclic peptides that would be easier to purify, simplify chemical synthesis and increase the yield of the multiantigenic peptide (MAP).

In the first process, PEP1 preparations were modified at the termini, also to eliminate Cys (labeled Mod 1 **[7]**, Mod 2 **[8]**, Mod 3 **[9]** and Mod 4 **[10]** [**Table S1e**]) and tested for comparative antigenicity using mAb 2C7 as the probe. Mod 3, the most antigenic, was used to make 4 different Tetra-MAP derivatives (Tetra MAP 3.1 **[11]**, TetraMAP 3.2 **[12]**, TetraMAP 3.3 **[13]** and TetraMAP 3.4 **[14]**) (**Table S1f**) each of which, used singly, was poorly immunogenic in BALB/c mice (**Table S1g**; TetraMAP 1.1 **[3]** and TetraMAP 1.2 **[4]** were also tested for immunogenicity). Two linear concatemers of Mod 3, containing 2 copies of the nominal peptide using different length spacers were also non-immunogenic (**Table S1h, [15]** and **[16]**).

A second approach to conjugate the peptide mimitope (cyclic) to the core using Click chemistry (copper-catalyzed reaction of an azide with an alkyne to form a 5-membered heteroatom ring) was then used. Several cyclic monomeric mimotope peptides were constructed (Click pep#1 through Click pep#4; **Table S1i** and **[17]**and **[18]**) and used in the Click chemistry approach to couple them to Click Core 4 (**Table S1i** and **Table S1j**) but this approach yielded low amounts of product with excess residual substrate (**Table S1j; [17]** and **[18]**); this approach was therefore abandoned.

Finally, use of a reverse MAP core, where conjugation of monomers (**[19]**, **[20]** and **[21]**) was carried out through the C-terminus of the core structure. One of the preparations, Tetra-MAP Cyclic Peptide 2 (TMCP2) **[22]**, was modified at the N-terminus of the monomeric pepide (PEP1) so a stable non-reducible (covalent) thioether bond formed a cyclized peptide with the C-terminal Cys of the peptide (**Table S1k**). The two other peptides, TMCP3 **[23]** and TMCP4, **[24]** each contained internal disulfide bonds when cyclized leading, potentially, to the formation of heterogenous molecules

(**Table S1k**). TMCP3 bound best to mAb 2C7 as determined by inhibition ELISA (**Fig. S1A**). TMCP2 and TMCP3 yielded higher anti-LOS titers than TMCP4 in immunization experiments that used the tetrapeptides plus Sigma MPL adjuvant (**Fig. S1B**). Anti-TMCP2 antisera showed maximal complement-dependent bactericidal activity against *N. gonorrhoeae* strain 15253 in immunized mice (**Fig. S1C**) and was chosen for further development. It is worth noting that affinity of binding to mAb 2C7 did not correlate with the functional antibody response. In light of its stability and performance in functional studies, TMCP2 was chosen as the lead candidate and was characterized further.
