# Supplementary material for: Preclinical Efficacy of a Lipooligosaccharide Peptide Mimic Candidate Gonococcal Vaccine
Source: mBio. 2019 Nov 5;10(6):e02552-19. doi: 10.1128/mBio.02552-19 (PMC6831779; doi:10.1128/mBio.02552-19)
Supplement: FIG S1 [file mBio.02552-19-sf001.pdf]

Supplemental Figures

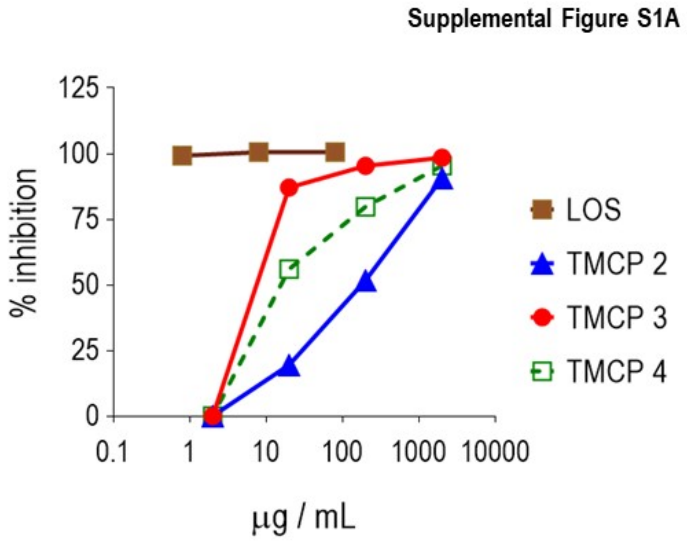

Fig. S1A. Inhibition of mAb 2C7 binding to solid phase-affixed (coated) gonococcal LOS by: TMCP2, TMCP3, TMCP4 (see Supplemental Text and Table S1k for description of peptides) and nominal LOS (control). mAb 2C7 (0.04 μg/ml) was added to microtiter wells coated with LOS purified from gonococcal strain 15253 in the presence of increasing concentrations of TMCP2, TMCP3, TMCP4 or LOS (positive control for 100% inhibition). The Y-axis shows the % inhibition of mAb 2C7 binding (residual binding) to immobilized LOS in the presence of the peptide (TMCP2 or CP2) or soluble LOS relative to binding of mAb 2C7 alone to immobilized LOS.

Supplemental Figure S1B

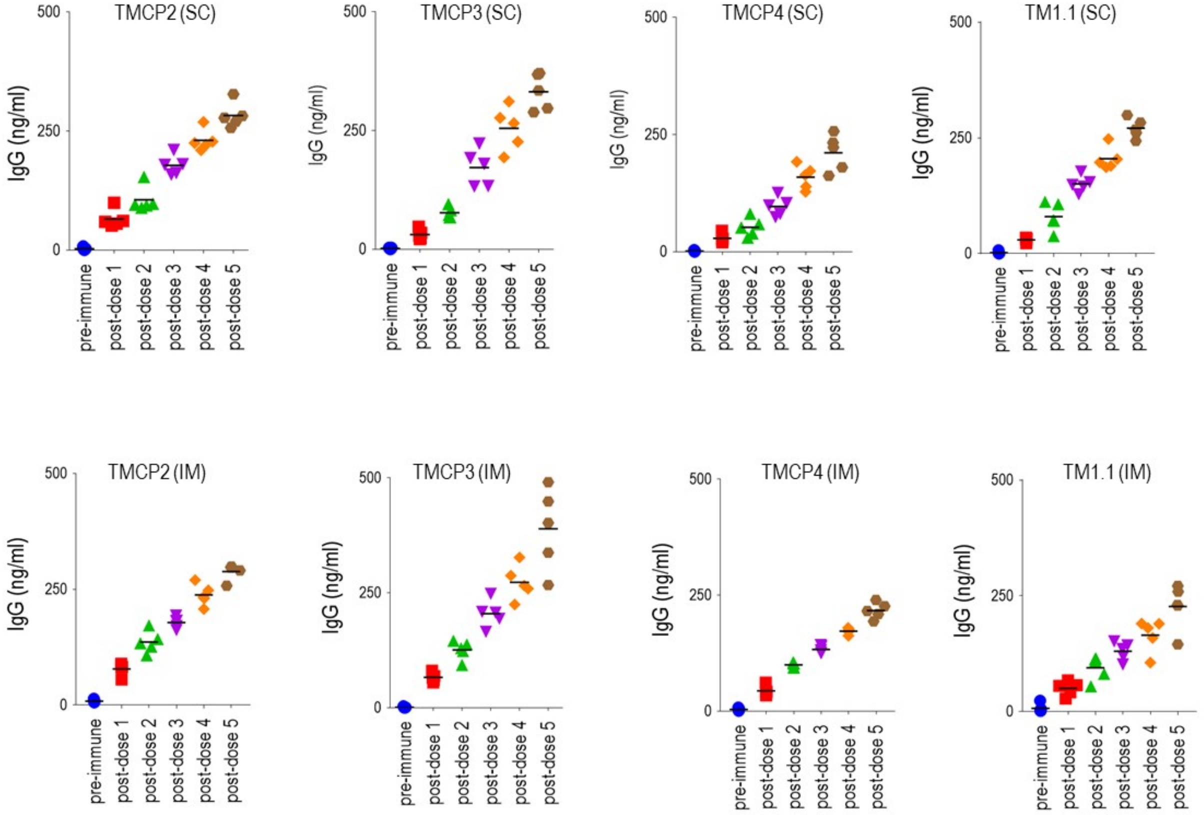

Fig. S1B. Anti-LOS antibody elicited by immunization of BALB/c mice with TMCP2, TMCP 3 and TMCP4 (see Supplemental Text and Table S1k for description of peptides). Antibodies elicited against LOS purified from *N. gonorrhoeae* strain 15253 (2C7-positive) in mice (n=5/group) immunized with tetra-MAP vaccine candidates TMCP2, TMCP3 and TMCP4 (50 µg/dose at weeks 0, 3, 6, 9 and 12) and Sigma MPL adjuvant given via the subcutaneous (SC) or intramuscular (IM) routes were measured by ELISA. A similar immunization schedule had been used previously with Octa-MAP1 (13) and was undertaken to permit development of maximal antibody titers. Tetra-MAP1.1 has the same peptide sequence and poly-lysine core ((Lys)$_2$Lys-β-Ala-COOH) described previously (19) and was used as a control. Sera collected from each mouse

at weeks 0 (pre-immune), 2 (post-dose 1), 5 (post-dose 2), 8 (post-dose 3), 11 (post-dose 4) and 14 (post-dose 5) were tested for reactivity with 15253 LOS.

Supplemental Figure S1C

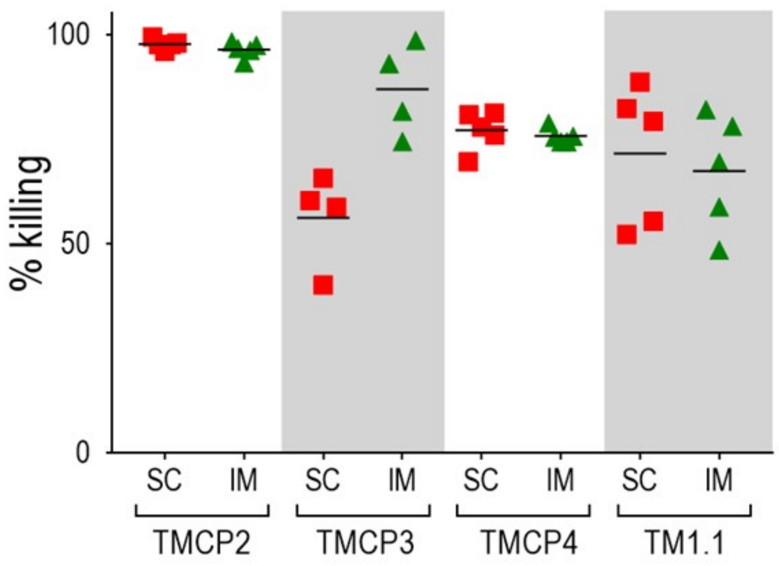

Fig. S1C. Serum bactericidal activity (SBA) of mouse antisera raised against tetra-MAP vaccine candidates (50 µg/dose at weeks 0, 3, 6, 9 and 12) with Sigma MPL adjuvant given via the subcutaneous (SC) or intramuscular (IM) routes (see Fig. S2 for immunization details). See Supplemental Text and Table S1 for a description of peptides. Post-dose 5 mouse anti-serum (IgM depleted) was tested against *N. gonorrhoeae* strain 15253 at a final dilution of 1:3 in the presence of 17% normal human serum (NHS) as the complement source. Y-axis, % killing ([CFU at t_0 – CFU at t_{30} / CFU at t_0] x 100).

Supplemental Figure S1D

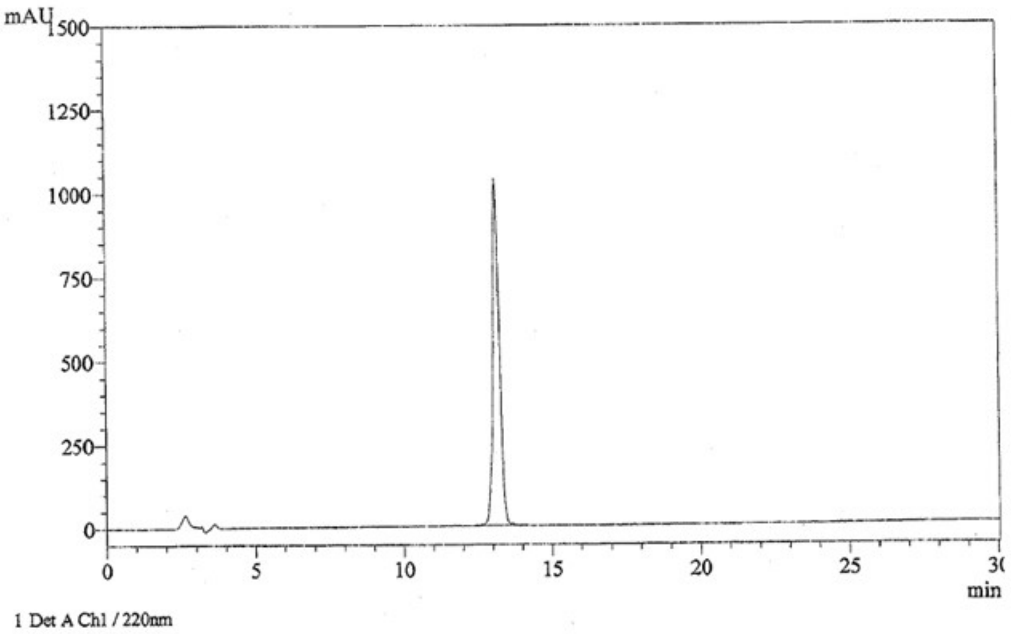

PeakTable

Detector A Ch1 220nm

Ret. Time	Area	Height	Area %
11.330	9220	1134	0.055
11.683	27987	1788	0.167
11.897	25373	2023	0.151
12.733	44185	5677	0.263
13.155	16628690	1037985	99.001
13.600	50018	5052	0.298
21.041	11063	1302	0.066
			100.000

Fig. S1D. The purity of TMCP2 determined by high-performance reverse-phase separation. TMCP2 was analyzed using a YMC-Pack-ODS-A C18 column (4.6 mm x 250 mm) with a particle size of 5 µm and pore size of 200 Å using a linear gradient of 0.05% trifluoroacetic acid (TFA) versus acetonitrile (MeCN) as the nonpolar solvent at a flow rate of 1 ml/min. A major peak (99% of the total area) at 13.155 min was noted.

Supplemental Figure S1E

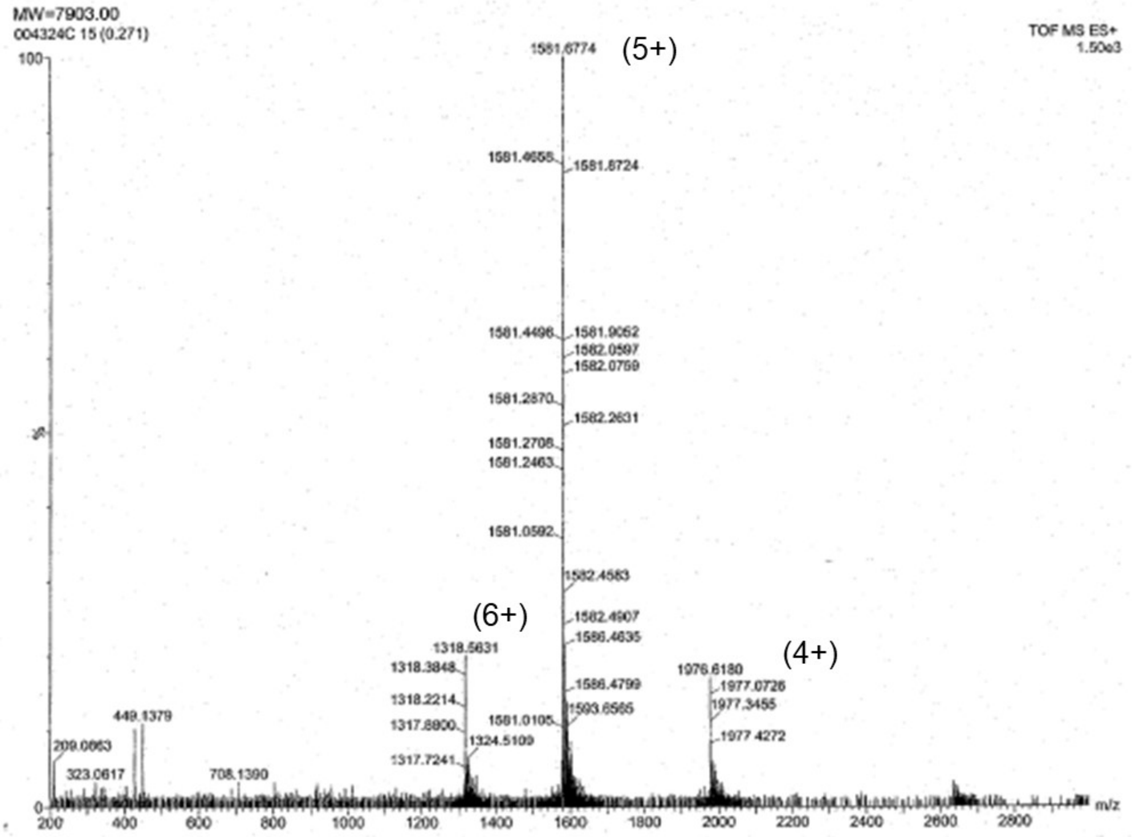

Fig. S1E. Positive electrospray ionization time-of-flight mass spectrometry (TOF MS ES+) of purified TMCP2. The TMCP-2 tetra MAP peptide was shown to be highly homogeneous by both analytical RP-HPLC as well as ESI-MS where the multicharged ions at M+4 (m/z=1976.62), M+5 (m/z=1581.68) and M+6 (m/z=1318.56) supported the validity of this conclusion.
