# Supplementary material for: Preclinical Efficacy of a Lipooligosaccharide Peptide Mimic Candidate Gonococcal Vaccine
Source: mBio. 2019 Nov 5;10(6):e02552-19. doi: 10.1128/mBio.02552-19 (PMC6831779; doi:10.1128/mBio.02552-19)
Supplement: TABLE S1 [file mBio.02552-19-st001.pdf]

Table S1. Summary of peptides synthesized and tested

**Table S1a**	
**Original Tetra- and Octa-MAP constructs**	
<u>Monomeric mimitope sequence (PEP1)</u>: CGP**IPVLDENGLFAP**GPC (aa's in bold red font) indicates the consensus sequence derived from a peptide display library; N- and C-terminal CGP and GPC, respectively, were retained from the FliC scaffold in the pFliTrx™ system, to facilitate circularization, if needed *(Ngampasutadol J et al. Vaccine. 2006.24(2):157-170; Gulati S et al. PLoS Pathog. 2013.9(8):e1003559)*<br><br><u>Tetra MAP 1</u><br>(CGP**IPVLDENGLFAP**GPC)$_4$(Lys)$_2$Lys-β-Ala-COOH<br><br><u>Octa MAP 1</u><br>(CGP**IPVLDENGLFAP**GPC)$_8$(Lys)$_4$(Lys)$_2$Lys-β-Ala-COOH	'Stepwise' addition of amino acids to poly-lysine core. The resulting product was too heterogenous for clinical development (Octa MAP 1 product shown below) and was abandoned.<br>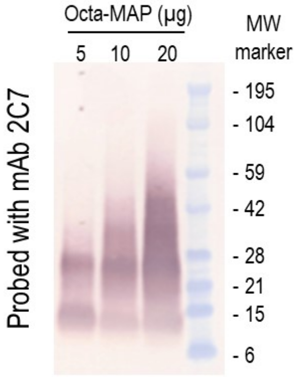
**Table S1b**	
**Tetra-MAP linked to Poly-Lys-maleamide core**	
<u>Peptide</u>: Ac-(CGP**IPVLDENGLFAP**GPC)-Lys-OH<br>Cys residues linked by disulfide<br><br><u>Core</u>: β-Ala-Lys-(Lys)$_2$-(Mal)$_4$	The product was too heterogenous and this approach was abandoned.
**Table S1c**	
**Modular addition of mimitope PEP1 to poly-Lysine core**	
<u>Octa-MAP1-modular</u><br>[H$_2$N-Cys(StBu)GP**IPVLDENGLFAP**GP(CysMAL-PEG$_4$)-amide]$_8$(Lys$_7$MAP)(bA)-amide	Mimitope (PEP1) added 'en bloc' to poly-lysine core<br>A modular system approach where each 18 aa single peptide (PEP1) was synthesized first and then purified to homogeneity. The N-terminal Cys was protected with a tert-butylsulfinyl (StBu) group; -SH of the C-terminal cysteine was linked to a heterobifunctional cross-linker intermediary molecule (MAL-PEG$_4$-NHS), through the maleimide (MAL) reactive group at one end. By displacing the reactive N-hydrosuccinimide (NHS) on the other end, PEP1-MAL-PEG molecules were amide-linked to each of 8 Lys residues of the Poly-Lys backbone. PEG$_4$ was included to create a water-soluble product. After synthesis and purification, the final octa-MAP product is treated with 0.5M tris-(2-carboxyethyl)phosphine hydrochloride to remove StBu from the N-terminal cysteine. This octa-MAP (~ 20.3 kDa) contained >90% complete octa-MAP. The process was too inefficient (low yield) for scale-up production of an economical vaccine product and therefore abandoned.

Table S1d

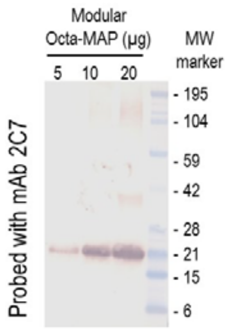

TetraMAPs without terminal Cys residues

Table S1e

Monomeric (circularized) peptides used for construction of tetra-MAPs (using modular approach)

Mod1: SGP**IPVLDENGLFAP**GPS<br><br>Mod2: **IPVLDENGLFAP**-PEG_2-KK<br><br>Mod3: K**IPVLDENGLFAP**-PEG_2-KK<br><br>Mod4: SG**PIPVLDENGLFAP**-PEG_2-KK	This set of peptides (PEP1s) was constructed without terminal Cys residues because: 1) the oxidation state of the Cys residues was uncertain – i.e., it was not clear if all peptides were in a given state (monomers, linear, cyclic) with respect to the sulfhydryl groups and 2) the tetra-MAP construction (reviewed above) had utilized 4 maleamide residues from a Lys dendrimer core that reacted both with primary amines and sulfhydryl groups. These factors likely contributed to product heterogeneity of the original Octa- and Tetra-MAPs<br><br>The circular monomeric peptides were tested for their ability to inhibit mAb 2C7 binding to LOS in order to down-select for further tetra-MAP synthesis. PEP1 is the original mimitope peptide monomer. Mod3 showed the best ability to inhibit mAb 2C7 binding to LOS (inhibition ELISA assay described in methods) and was chosen to generate tetra-MAP derivates 3.1, 3.2, 3.3 and 4.4 (described below; **Table S1f**)

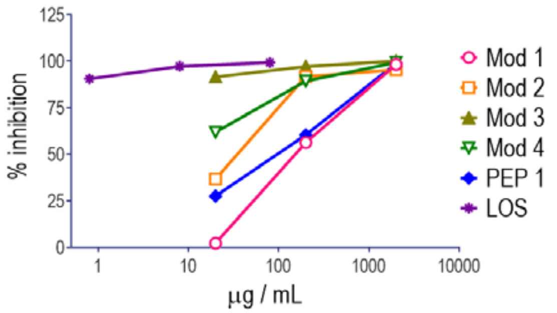

Table S1f

Tetra-MAP derivatives of Mod3 (dendrimers)

TetraMAP3.1<br>(K**IPVLDENGLFAP**-PEG_2-KK)$_4$-MAP_4<br><br>TetraMAP3.2<br>(K**IPVLDENGLFAP**-PEG_2-KKC)$_4$-Maleimide$_4$-MAP_4	Mod3 was linked to different core structures as described on the left. Immunogenicity was evaluated in BALB/c mice (n=5 mice/ group). IgG against 2C7$^+$ LOS was measured by ELISA. Original Octa MAP1 and TetraMAP1 compounds were included as historical (positive) controls. (shown below in **Table S1g**). All Mod3 derivatives gave poor responses.

TetraMAP3.3
K**IPVLDENGLFAP**-D Lys-D Lys$_4$-K$_2$-K-MAP$_4$

TetraMAP3.4
K**IPVLDENGLFAP**GPC-D Lys-D Lys$_4$-K$_2$-K-MAP$_4$
Three GPC residues at the C-terminus (also present in PEP1) were added to Mod3 to examine their role in immunogenicity

Additional tetra-MAP peptides synthesized:

Tetra MAP1.1: control peptide; identical structure to original Tetra-MAP1, but made by modular synthesis

(CGP**IPVLDENGLFAP**GPC)$_4$(Lys)$_2$Lys-β-Ala-COOH

Tetra MAP1.2: N-terminal Cys in TetraMAP1.1 replaced with Ser in attempt to reduce unwanted disulfide bond formation

SGP**IPVLDENGLFAP**GPC)$_4$(Lys)$_2$Lys-β-Ala-COOH

Immunization studies (shown on the right; **Table S1g**), in addition to using original stepwise synthesized antigens (Octa MAP1 and Tetra MAP1) and MOD3 derivatives for immunization, also included immnizations with Tetra MAP 1.1 and Tetra MAP 1.2 (n=9 mice/group). TetraMAP1.1 (bottom row) elicited similar anti-LOS Ab levels as Tetra MAP1 (top row).

Table S1g

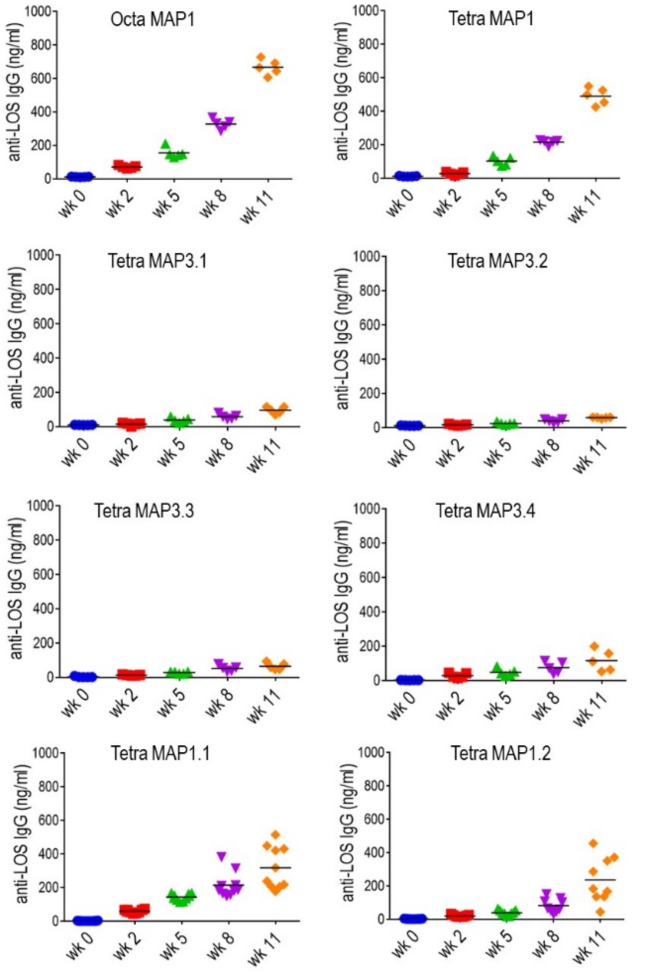

Table S1h

Concatemers (dimers) derived from Mod3 sequence

Concatemers (dimers) derived from Mod3 sequence

Mod3 linear dimer #1:
K**IPVLDENGLFAP**GSK**IPVLDENGLFAP**

Mod3 linear dimer #2:
K**IPVLDENGLFAP**AAAGGK**IPVLDENGLFAP**

Linear peptides were studied in 2C7 inhibition ELISA – the molecule with AAAGG linker inhibited mAb 2C7 binding to LOS better than the molecule with the AG linker (not shown), but neither were immunogenic in mice (shown below).

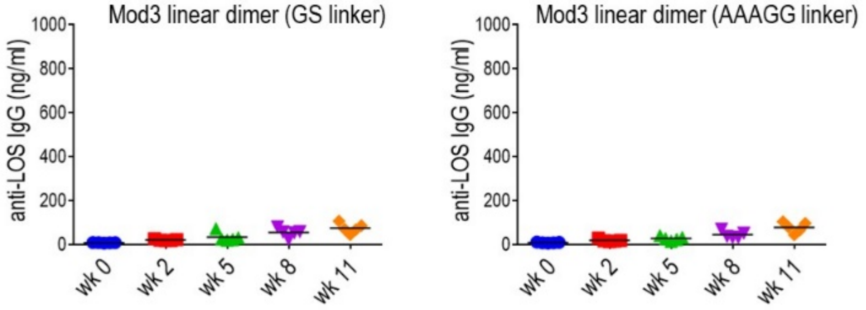

**Table S1i**	
**Cyclic peptides and Core synthesized for Click chemistry linkage**	
Click pep#1:<br>Cyclic H-(CGPIPVLDENGLFAPGPC)-K-alkyne<br><br>Click pep#2:<br>Cyclo H-(AGPIPVLDENGLFAPGPC)-K-alkyne<br><br>Click pep#3:<br>Cyclic Ac-(CGPIPVLDENGLFAPGPC)-K-OH<br><br>Click pep#4:<br>Cyclic GP(CIPVLDENGLFAPC)-GP-K-alkyne (synthesized to increase distance of Cys from Core)<br><br>Click Core 4:<br>(6-Azido-hexynoyl)$_4$-Lys$_2$-Lys-β-Ala-OH (***structure A*** in Fig below; ***structure B*** represents expected product with Cyclic peptide #1)	Attempts to "click" the monomeric disulfide constrained peptides to the MAP Click-core 4 (6-Azido-hexynoyl)$_4$-Lys$_2$-Lys-β-Ala-OH (tetraMAP azide)) were minimally successful. RP-HPLC chromatography of the product after the reaction between the MAP Click-core showed only a small formation of the desired product with a large excess of residual substrate. Additional heterogeneity likely was caused by reduction of the disulfide bonds. This approach was abandoned.

Table S1j

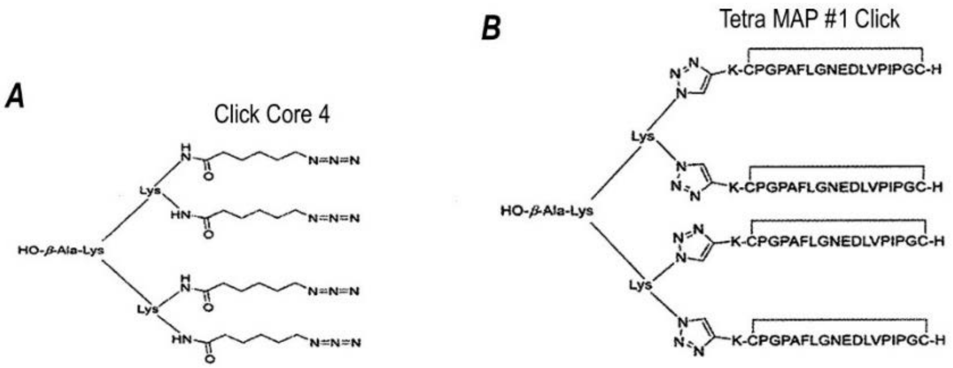

**Table S1k**	
**Reverse MAP constructs**	
TMCP2:<br>CH$_2$-CO-**GP**IPVLDENGLFAP**GPC**)-**K**-OH<br>MAP construct is made up of 4 individual peptides (peptide shown above) that each contain ***stable non-reducible thioether bonds*** for cyclization; cyclized peptides have no free –SH to form inter-molecular bonds. C-terminus is linked to reverse MAP core<br><br>TMCP3:<br>Ac-**CGP**IPVLDENGLFAP**GPC**-**K**-OH<br>MAP construct is made up of 4 individual peptides (peptide shown above) that contain	Reverse MAP constructs were developed as an inverse to the standard lysine branch approaches. Because each of the peptides were designed to contain only a single amine functional group, the development of a dendrimeric structure based upon a glutamic acid came about as a logical extension of this technology. The need for developing a combination solid-phase and solution process would help ensure a highly reproducible strategy, which would yield homogenous products. The key step in generating the tetraMAP was controlled by using a solution phase coupling step of highly purified monomers to the reverse tetra-MAP core. The reverse

internal S-S (disulfide) formed by the two cysteines (C); cyclized peptides have no free terminal –SH to form inter-molecular bonds but the internal S-S bonds are potentially reducible<br><br>TMCP4:<br>Ac-**GP**-(**C**IPVLDENGLFAP**C**)-**GPK**-OH<br>Same as TMCP3, but Cys at C-terminus was moved proximal (N-terminal) to the GP in order to move the cyclic peptide further away from the core.<br><br>Reverse MAP core:<br>Ac-N-β-Ala-Glu-$(Glu)_2$-$(COOH)_4$	MAP core construct starting with the Ac-N-β-Ala-Glu-$(Glu)_2$-$(COOH)_4$ was designed to position four carboxyl groups for modification with amine components from the peptide monomers.<br><br>Inhibition ELISA assay – see **Fig. S1**<br><br>Immunogenicity (IgG responses elicited in BALB/c mice) – see **Fig. S2**<br><br>Serum bactericidal activity of immune sera – see **Fig. S3**<br><br>***Based on manufacturability, its stable thioether bond for cyclization and elicited bactericidal activity, TMCP2 was selected as the lead candidate for further development.***
