# Supplementary material for: Preclinical Efficacy of a Lipooligosaccharide Peptide Mimic Candidate Gonococcal Vaccine
Source: mBio. 2019 Nov 5;10(6):e02552-19. doi: 10.1128/mBio.02552-19 (PMC6831779; doi:10.1128/mBio.02552-19)
Supplement: TABLE S2 [file mBio.02552-19-st002.pdf]

Table S2. Chemical structures of peptides tested

#	Name		Structure	Linker	MAP Core
**1**	Tetra MAP 1		(CGP**IPVLDENGLFAP**GPC)$_4$	No linker	(Lys)$_2$Lys-β-Ala-COOH<br>(B-Ala = β-Ala)
**2**	Octa MAP 1		(CGP**IPVLDENGLFAP**GPC)$_8$	No linker	(Lys)$_4$ (Lys)$_2$ Lys-β-Ala-COOH
**3**	Tetra MAP 2<br>(Control)		(CGPPEARDEGTITLERGPC)$_4$	No linker	(Lys)$_2$Lys-β-Ala-COOH
**-**	Tetra MAP 1.1	Api 1875	(CGP**IPVLDENGLFAP**GPC)$_4$		(Lys)$_2$Lys-β-Ala-COOH
**4**	Tetra MAP 1.2	Api 1876	(SGP**IPVLDENGLFAP**GPC)$_4$		(Lys)$_2$Lys-β-Ala-COOH
**5**	Octa-MAP	Api 1873	H_2N-C(StBu)GP**IPVLDENGLFAP**GP(CysMal-PEG$_4$)-amide]$_8$(Lys$_7$MAP)(bA)-amide	Ordered but unable to produce	
**6**	Octa-MAP	Api 1874	H_2N-SGP**IPVLDENGLFAP**GP(CysMal-PEG$_4$)-amide]$_8$(Lys$_7$MAP)(bA)-amide	Ordered but unable to produce	
**7**	Mod 1	903482	SGP**IPVLDENGLFAP**GPS	N/A	
**8**	Mod 2	903483	**IPVLDENGLFAP**	-PEG$_2$-KK)-	
**9**	Mod 3	903484	K**IPVLDENGLFAP**	-PEG$_2$-KK)-	
**10**	Mod 4	903485	SGP**IPVLDENGLFAP**	-PEG$_2$-KK)-	
**11**	TMAP 3.1	904680	K**IPVLDENGLFAP**	-PEG$_2$-KK)-	-MAP$_4$
**12**	TMAP 3.2	904681	K**IPVLDENGLFAP**	-PEG$_2$-KKC)-	$_4$-Maleimide$_4$-MAP$_4$
**13**	TMAP 3.3	906164	K**IPVLDENGLFAP**<br>**(K=Lys)**	-D-Lys-D-Lys$_4$<br>**'D-Lys' is the dextro- form of Lys.**	-K$_2$-K-MAP$_4$
**14**	TMAP 3.4	906165	K**IPVLDENGLFAP**GPC	-D-Lys-D-Lys$_4$	-K$_2$-K-MAP$_4$
**15**	Mod 3 dimer #1		K**IPVLDENGLFAP**GSKK**IPVLDENGLFAP**	Concatemer	
**16**	Mod 3 dimer #2		K**IPVLDENGLFAP**AAAGGKK**IPVLDENGLFAP**	Concatemer	
**17**	PCS-32325-PI	Cyclized peptide	cyclic-CGP-**IPVLDENGLFAP**-GPC-K-alkyne*<br>this is a disulfide constrained **monomer**<br>(K= Lys)<br>*(5-Hexynoyl)-OH	(N_3K$_2$-K-B-Ala-OH)*<br>*There is no core because the structure is a disulfide constrained monomer: see the attached information sheet (PCS-32325-PI)<br>(click chemistry was not successful)	
**18**	PCS-32328-PI	Cyclized peptide	cyclic-GPC-**IPVLDENGLFAP**-CGP-K-alkyne*	(N_3K$_2$-K-B-Ala-OH)<br>(click chemistry was not successful)	

**19**	PCS-32403-PI	Cyclic Peptide 1<br>CP1	cyclic Ac-CGP-**IPVLDENGLFAP-**GPC-K-OH<br>Same as cyclic peptide 3 (CP3)		Tested as a monomer
**20**	PCS-32404-PI	Cyclic Peptide 2<br>CP2	(CH2-CO-GP**IPVLDENGLFAP**GPC)-K-OH		Tested as a monomer
**-**	PCS-32327-PI	Cyclic Peptide 3<br>CP3	cyclic Ac-CGP-**IPVLDENGLFAP-**GPC-K-OH<br>Same as cyclic peptide 1 (CP1)		Tested as a monomer
**21**	PCS-32405-PI	Cyclic Peptide 4<br>CP4	Ac-GP-(C**IPVLDENGLFAP**C)-GP-K-OH		Tested as a monomer
**22**	PCS-32330-PI	Tetra MAP<br>Cyclic Peptide 2;<br>TMCP2 reverse	[(CH2-CO-GP**IPVLDENGLFAP**GPC)-K-OH]$_4$		Glu_2-Glu-βAla-N-Ac<br>-NH_2 group of the K (Lys) links to the -COOH group of Glu "reverse linkage")
**23**	PCS-32331-PI	Tetra MAP<br>Cyclic Peptide 3;<br>TMCP3 reverse	[Ac-(CGP**IPVLDENGLFAP**GPC)-K-OH]$_4$		Glu_2-Glu-βAla-N-Ac<br>"reverse linkage
**24**	PCS-32332-PI	Tetra MAP<br>Cyclic Peptide 4;<br>TMCP4 reverse	[Ac-GP-(C**IPVLDENGLFAP**C)-GP-K-OH]$_4$		Glu_2-Glu-βAla-N-Ac<br>"reverse linkage"
**-**	PCS-32329-PI		[Ac-(CGP**IPVLDENGLFAP**GPC)-K-OH]$_4$<br>Duplicate of PCS-32331		
