# Supplementary material for: Preclinical Efficacy of a Lipooligosaccharide Peptide Mimic Candidate Gonococcal Vaccine
Source: mBio. 2019 Nov 5;10(6):e02552-19. doi: 10.1128/mBio.02552-19 (PMC6831779; doi:10.1128/mBio.02552-19)
Supplement: FIG S2 [file mBio.02552-19-sf002.pdf]

Supplemental Figure S2

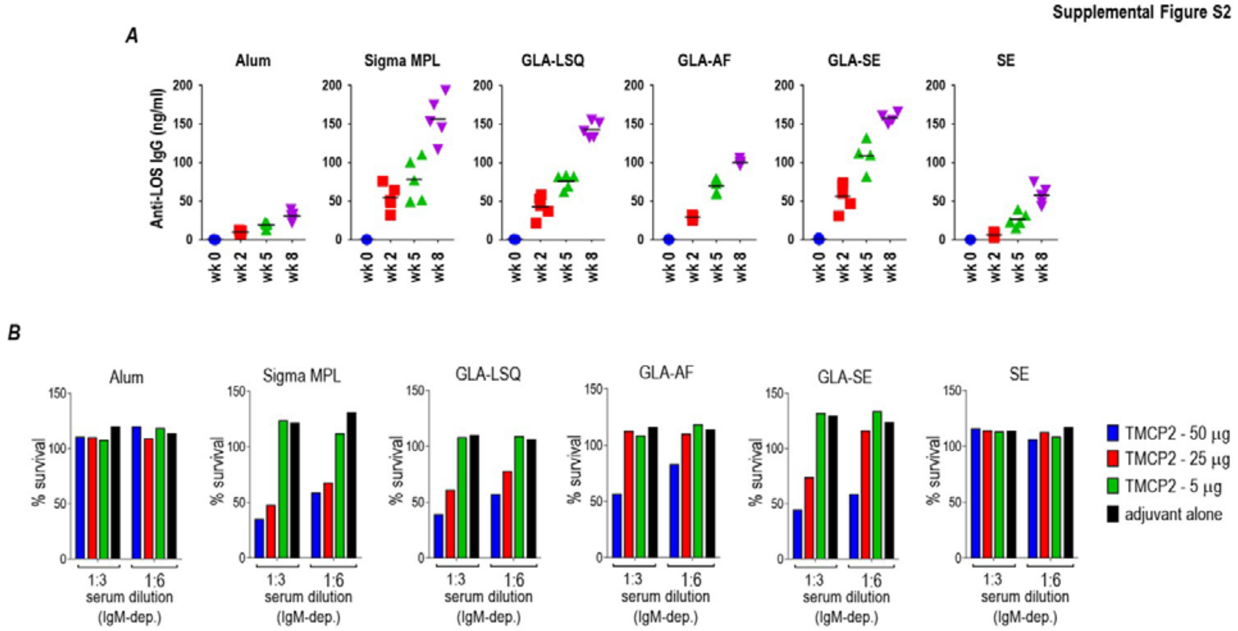

Fig. S2. Choice of GLA-SE as the adjuvant for tetra-MAP vaccine candidate TMCP2. ***A***. Immunogenicity of TMCP2 with different adjuvants. BALB/c mice (n=5/group) were immunized IM with 5, 25 or 50 µg of TMCP2 plus the adjuvants indicated above each graph at 0, 3 and 6 weeks. Pre-immune sera (wk 0) and sera collected 2 weeks after each dose (wk 2, 5 and 8) were assayed for IgG against *Ng* 15253 LOS by ELISA. Data using the 50 µg dose, which elicited the highest responses, are shown above. ***B***. Serum bactericidal activity antisera elicited by TMCP2 immunization with different adjuvants. Week 8 (post-dose 3) antisera from all 5 mice in each group were pooled and tested in serum bactericidal assays at anti-serum dilutions of either 1:3 or 1:6, with normal human serum (NHS) as the complement source. Because intact antisera (IgM replete) in all groups, including the adjuvant control groups, supported bactericidal activity (>95% killing in every instance (data not shown), the data shown above are following IgM depletion.
