# Supplementary material for: Preclinical Efficacy of a Lipooligosaccharide Peptide Mimic Candidate Gonococcal Vaccine
Source: mBio. 2019 Nov 5;10(6):e02552-19. doi: 10.1128/mBio.02552-19 (PMC6831779; doi:10.1128/mBio.02552-19)
Supplement: TABLE S3 [file mBio.02552-19-st003.pdf]

Supplemental Table S3. Bactericidal activity of naïve mouse sera against *N. gonorrhoeae* FA1090

Mouse strain	Source	Length of time housed at UMass	Titer of heat-inactivated mouse serum used in serum bactericidal assay	Number of animals with bactericidal activity [A] (%) versus FA1090
BALB/c	Charles River	<2 days	1:6	5/8 (63%)
BALB/c	Jackson	>4 months	≥1:6	4/4 (100%)
BALB/c	Jackson (parent)	Bred at UMass for 3 generations	≥1:3	5/5 (100%)
BALB/c	Envigo	<2 days	1:6	9/9 (100%)
BALB/c	Taconic	<2 days	1:15	3/5 (60%)
CMAH$^{-/-}$ (BALB/c background)	Univ. of California, San Diego	Bred for >5 generations	≥1:3	2/2 (100%)
C3$^{-/-}$ (C57BL6 backcrossed into BALB/c background)	Jackson (parent)	Bred at UMass for >8 generations	≥1:3	1/3 (33%)
Rag1$^{-/-}$ (BALB/c background)	Jackson (parent)	Bred at UMass for >5 generations	≥1:3	0/5 (0%)
JhD (BALB/c background)	Jackson (parent)	Bred at UMass for >5 generations	1:15	0/1 (0%)
JhD$^{+/-}$ (BALB/c background)	Jackson (parent)	Bred at UMass for >5 generations	1:15	2/2 (100%)

[A] Bactericidal activity is defined as ≤50% survival following 30 min of incubation with 17% normal human serum (NHS) as the complement source.
