# Supplementary material for: Preclinical Efficacy of a Lipooligosaccharide Peptide Mimic Candidate Gonococcal Vaccine
Source: mBio. 2019 Nov 5;10(6):e02552-19. doi: 10.1128/mBio.02552-19 (PMC6831779; doi:10.1128/mBio.02552-19)
Supplement: TABLE S4 [file mBio.02552-19-st004.pdf]

Table S4A. Complement-dependent killing of *N. gonorrhoeae* FA1090 in intact and IgM-depleted immune sera (data displayed graphically in Fig 2B)

Mouse #	Survival in intact immune serum [A] % (dilution) of immune serum			Survival in IgM-depleted immune serum [A] % (dilution) of immune serum		
	3.3% (1/30)	6.7% (1/15)	16.7% (1/6)	3.3% (1/30)	6.7% (1/15)	16.7% (1/6)
**50 µg/dose**						
50-1	81.13	71.50	49.24	89.15	76.53	53.71
50-2	21.21	15.00	9.95	29.30	21.78	15.00
50-3	16.59	10.71	1.90	19.91	14.93	3.41
50-4	16.33	5.43	0.00	22.03	17.81	2.24
50-5	2.84	0.47	0.00	5.38	2.33	0.00
**100 µg/dose**						
100-1	11.43	0.52	0.00	16.67	4.57	0.90
100-2	35.42	22.48	5.21	47.17	18.34	11.42
100-3	2.54	0.48	0.47	8.07	0.89	0.46
100-4	59.60	44.19	33.33	76.23	61.40	48.89
100-5	64.82	46.30	27.41	76.44	50.00	34.51
**200 µg/dose**						
100-1	9.81	5.21	0.00	9.61	4.89	1.79
100-2	26.39	0.93	0.00	33.64	2.28	1.93
100-3	2.00	0.00	0.00	3.56	1.91	0.00
100-4	74.88	43.12	6.19	85.02	54.50	10.73
**Adj. cont.**						
A-1	ND [B]	ND	108.75	ND	ND	114.29
A-2	ND	ND	109.47	ND	ND	111.59
A-3	ND	ND	109.58	ND	ND	111.66
A-4	ND	ND	110.79	ND	ND	116.63
A-5	ND	ND	109.31	ND	ND	113.27

[A] Normal human serum (16.7%) was used as the complement source
[B] ND; not done

Table S4B. Complement-dependent killing of *N. gonorrhoeae* FA1090 in intact and IgM-depleted immune sera (data are displayed graphically in Fig 3B)

Mouse #	Survival in intact immune serum [A]			Survival in IgM-depleted immune serum [A]		
	% (dilution) of immune serum			% (dilution) of immune serum		
	10% (1/10)	12.5% (1/8)	16.7% (1/6)	10% (1/10)	12.5% (1/8)	16.7% (1/6)
**50 µg/dose**						
50-1	48.10	30.23	8.96	56.17	46.73	29.34
50-2	23.12	11.50	1.49	61.90	27.66	10.13
50-3	21.67	6.63	0.00	62.86	34.72	13.54
50-4	50.73	27.86	16.24	66.51	47.96	33.63
50-5	59.02	27.27	7.39	75.71	50.23	29.65
50-6	48.74	40.39	24.50	58.37	43.26	36.07
50-7	50.25	34.17	15.42	59.73	42.40	24.00
50-8	56.28	48.50	16.75	67.25	52.53	23.45
50-9	45.73	38.24	10.45	56.33	49.78	36.65
50-10	41.12	25.62	5.45	52.61	34.88	9.72
50-11	39.20	27.09	6.28	48.39	38.46	10.96
50-12	50.50	39.49	22.17	68.00	48.62	34.12
50-13	57.50	30.10	1.50	61.84	39.73	15.42
**100 µg/dose**						
100-1	49.25	10.55	3.05	58.41	26.11	20.20
100-2	21.43	14.50	8.59	30.00	25.58	20.87
100-3	23.90	10.10	3.78	31.75	14.93	9.33
100-4	52.04	29.35	19.39	55.72	42.47	30.14
100-5	31.07	14.14	1.00	39.05	18.55	2.79
100-6	37.31	4.85	0.50	60.00	21.43	1.67
100-7	20.29	9.14	4.02	27.23	19.38	15.15
100-8	65.85	49.75	15.27	78.74	59.15	23.24
100-9	31.12	15.27	0.00	48.79	28.40	3.14
100-10	36.36	8.76	0.00	57.21	26.27	2.39
100-11	33.50	10.71	4.04	39.04	20.27	10.92
100-12	23.15	3.98	0.50	56.28	20.35	2.16
100-13	40.40	16.99	2.45	53.11	25.11	2.78
**200 µg/dose**						
200-1	52.66	27.75	6.53	50.00	26.42	14.23
200-2	54.64	38.50	7.85	46.00	32.20	16.33
200-3	53.23	25.91	6.47	50.21	31.90	23.08
200-4	38.07	19.90	0.00	54.11	28.63	16.20
200-5	24.74	5.97	0.51	50.00	27.49	8.68
200-6	22.39	0.00	0.00	42.01	18.67	13.49
200-7	28.00	4.06	2.06	46.89	22.75	15.12
200-8	31.47	10.88	0.00	49.77	32.86	16.16
200-9	11.92	0.50	0.00	54.59	22.57	3.90
200-10	8.29	1.46	0.00	23.14	18.35	21.00
200-11	23.23	6.67	1.94	55.00	36.86	20.76
200-12	16.58	4.08	3.02	42.86	26.05	10.55
200-13	39.59	15.34	1.01	57.64	31.94	7.21
**Adj. control**						
A-1	ND [B]	ND	111.11	ND	ND	107.59
A-2	ND	ND	108.10	ND	ND	118.58
A-3	ND	ND	109.13	ND	ND	117.08
A-4	ND	ND	109.58	ND	ND	ND
A-5	ND	ND	109.21	ND	ND	ND
A-6	ND	ND	108.33	ND	ND	ND
A-7	ND	ND	110.37	ND	ND	ND
A-8	ND	ND	108.75	ND	ND	ND
A-9	ND	ND	112.03	ND	ND	ND
A-10	ND	ND	112.35	ND	ND	ND
A-11	ND	ND	110.25	ND	ND	ND
A-12	ND	ND	111.52	ND	ND	ND
A-13	ND	ND	111.43	ND	ND	ND

[A] Normal human serum (16.7%) was used as a source of complement; [B] ND; not done

Table S4C. Complement-dependent killing of *N. gonorrhoeae* MS11 in intact and IgM-depleted immune sera (data are displayed graphically in Fig 3C)

Mouse #	Survival in intact immune serum [A] % (dilution) of immune serum			Survival in IgM-depleted immune serum [A] % (dilution) of immune serum		
	1.3% (1/75)	3.3% (1/30)	6.7% (1/15)	1.3% (1/75)	3.3% (1/30)	6.7% (1/15)
**50 µg/dose**						
50-1	18.27	0.48	0.00	40.52	19.75	3.11
50-2	39.90	26.47	0.00	72.26	41.03	10.98
50-3	49.28	19.91	0.00	82.67	45.00	12.58
50-4	15.50	0.00	0.00	35.00	0.00	0.00
50-5	6.03	0.99	0.00	21.94	0.00	0.00
50-6	14.78	0.51	0.00	26.99	0.00	0.00
50-7	10.50	0.97	0.00	23.53	0.00	0.00
50-8	22.93	7.39	1.52	38.75	12.66	2.65
50-9	10.61	0.00	0.00	25.48	0.00	0.00
50-10	18.60	9.95	0.00	44.30	11.66	4.52
50-11	31.66	11.33	0.00	37.75	16.34	0.63
50-12	19.91	0.48	0.47	27.10	0.00	0.00
50-13	47.85	19.43	0.93	64.67	34.62	9.82
**Adj. cont.**						
A-1	ND	ND	108.53	ND	ND	109.38
A-2	ND	ND	113.59	ND	ND	112.99

[A] Normal human serum depleted of IgG and IgM (Human complement [Pel-Freez]) 6.7% was used as the source of complement

[B] ND; not done

Table S4D. Complement-dependent killing of *N. gonorrhoeae* FA1090 in intact and IgM-depleted immune sera (data are shown graphically in Fig 5C)

Mouse #	Survival in intact immune serum [A]			Survival in IgM-depleted immune serum [A]		
	% (dilution) of immune serum			% (dilution) of immune serum		
	10% (1/10)	12.5% (1/8)	16.7% (1/6)	10% (1/10)	12.5% (1/8)	16.7% (1/6)
**50 µg/dose**						
50-1	60.09	44.89	21.79	69.58	56.73	33.47
50-2	30.95	22.64	4.70	48.84	31.72	19.75
50-3	55.33	45.70	21.24	61.47	50.20	30.83
50-4	50.67	37.26	18.94	59.28	42.39	24.03
50-5	52.36	37.00	21.56	64.20	49.15	31.95
50-6	46.30	31.98	12.56	54.22	37.23	21.03
50-7	63.98	36.68	21.76	76.02	58.01	30.86
50-8	47.44	27.70	8.64	59.58	38.20	20.52
50-9	57.08	41.94	30.81	73.25	52.65	36.09
50-10	56.22	40.74	19.18	65.42	50.42	28.63
50-11	65.00	45.95	21.97	75.54	59.29	34.57
**Adj. cont.**						
A-1	ND [B]	ND	110.45	ND	ND	107.53
A-2	ND	ND	111.37	ND	ND	113.64
A-3	ND	ND	113.13	ND	ND	ND
A-4	ND	ND	107.50	ND	ND	ND
A-5	ND	ND	109.09	ND	ND	ND
A-6	ND	ND	110.00	ND	ND	ND
A-7	ND	ND	118.09	ND	ND	ND
A-8	ND	ND	112.44	ND	ND	ND
A-9	ND	ND	112.02	ND	ND	ND
A-10	ND	ND	114.72	ND	ND	ND
A-11	ND	ND	106.51	ND	ND	ND

[A] Normal human serum (16.7%) was used as a source of complement

[B] ND; not done
