# Supplementary material for: Preclinical Efficacy of a Lipooligosaccharide Peptide Mimic Candidate Gonococcal Vaccine
Source: mBio. 2019 Nov 5;10(6):e02552-19. doi: 10.1128/mBio.02552-19 (PMC6831779; doi:10.1128/mBio.02552-19)
Supplement: FIG S3 [file mBio.02552-19-sf003.pdf]

Supplemental Figure S3A

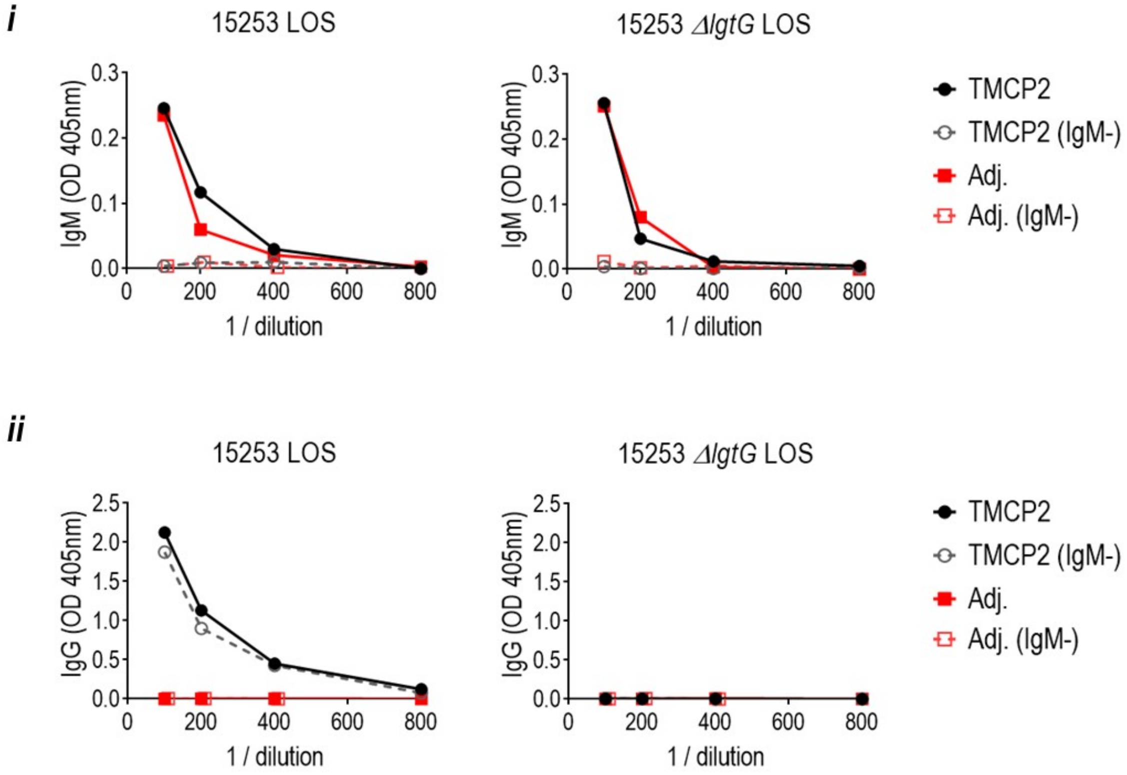

Fig. S3A. Reactivity of IgM in immune and adjuvant control sera against LOS and FA1090. Post-dose 3 sera from mice (n=13) immunized with TMCP2 (50 µg/dose) plus GLA-SE were pooled (labeled 'TMCP2'); one aliquot was immunodepleted of IgM by passage over anti-mouse IgM agarose ('TMCP2 (IgM-)'). Similarly, an aliquot of pooled adjuvant control sera ('Adj.') was depleted of IgM ('Adj. (IgM-)'). The intact and IgM-depleted sera were tested for reactivity against LOS purified from strain 15253 (2C7-positive) or 15253 *ΔlgtG* (2C7-negative). ***i.*** IgM binding LOS. ***ii***. IgG binding to LOS. X-axes, reciprocal serum dilution; Y-axes, antibody (IgM or IgG) binding (OD_{405nm}). Note the different Y-axes scales in the upper and lower panels.

Supplemental Figure S3B

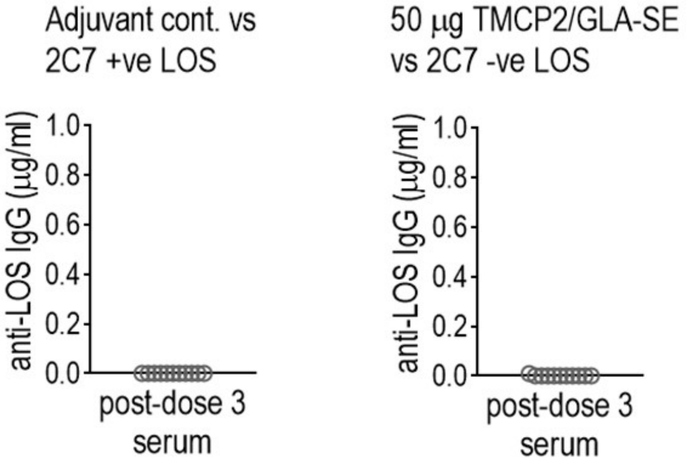

Fig. S3B. Specificity of IgG response to the 2C7 LOS epitope. Post-dose 3 sera from adjuvant (GLA-SE) control mice (experiment described in Fig. 5) that were not infected with *N. gonorrhoeae* were tested for IgG reactivity with 2C7-positive LOS (left graph). Similarly, sera from TMCP2/GLA-SE immunized mice not used for gonococcal challenge were tested for reactivity of IgG against 2C7-negative LOS derived from strain 15253 *ΔlgtG* (right graph).
