# Supplementary material for: Preclinical Efficacy of a Lipooligosaccharide Peptide Mimic Candidate Gonococcal Vaccine
Source: mBio. 2019 Nov 5;10(6):e02552-19. doi: 10.1128/mBio.02552-19 (PMC6831779; doi:10.1128/mBio.02552-19)
Supplement: TABLE S5 [file mBio.02552-19-st005.pdf]

Table S5. Serum and vaginal anti-LOS Ab levels in BALB/c mice immunized with TMCP2/GLA-SE

	TMCP2/GLA-SE (Mouse #)					GLA-SE (Mouse #)				
	1	***2***	***3***	***4***	***5***	***6***	***7***	***8***	***9***	***10***
**Serum anti-LOS IgG (µg/ml)**	3.63	3.79	3.66	3.44	3.28	0.12	0.10	0.11	0.12	0.07
**Vaginal lavage anti-LOS IgG (µg/ml) [B]**	0.030	0.044	0.040	0.027	0.028	0.001	0.002	0.001	0.003	0.002

[A] Mice were immunized with 50 µg TMCP2 plus 5 µg GLA-SE per dose, given IM at 0, 3 and 6 weeks and serum and vaginal swabs collected 2 weeks after the 3rd dose were analyzed.

[B] Vaginal swabs were eluted in saline and the antibody concentration per ml is indicated
