# Supplementary material for: Preclinical Efficacy of a Lipooligosaccharide Peptide Mimic Candidate Gonococcal Vaccine
Source: mBio. 2019 Nov 5;10(6):e02552-19. doi: 10.1128/mBio.02552-19 (PMC6831779; doi:10.1128/mBio.02552-19)
Supplement: FIG S4 [file mBio.02552-19-sf004.pdf]

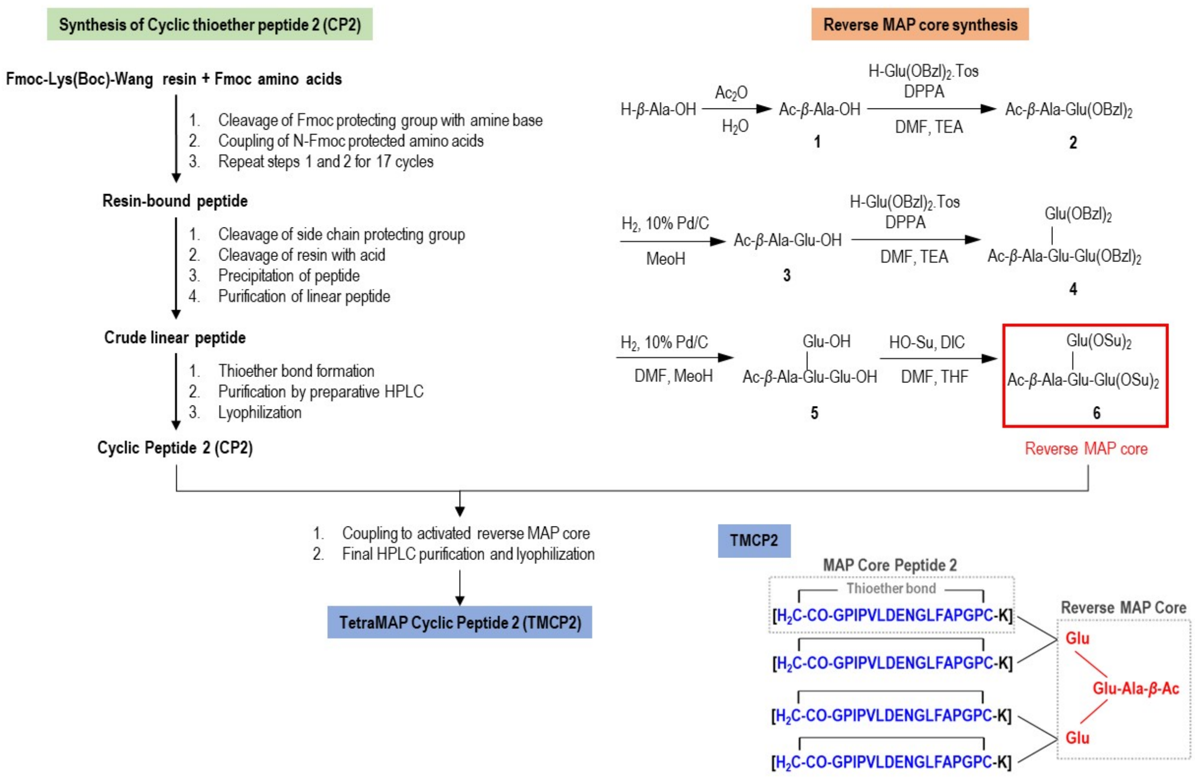

Fig. S4. Schematic summarizing the steps in the synthesis of TMCP2. MAP, multiantigen peptide; Ac_2O, acetic anhydride; DMF, N,N-dimethylformamide; DPPA, diphenylphosphoryl azide; Glu(OBzl), Glutamic acid 1-benzyl ester; TEA, triethylamine; HO-Su, N-hydroxysuccinimide; DIC, diisopropylcarbodiimide; Pd/C, palladium on carbon catalyst.
